# Integrative Strategies for Preventing and Managing Metabolic Syndrome: The Impact of Exercise and Diet on Oxidative Stress Reduction—A Review

**DOI:** 10.3390/life15050757

**Published:** 2025-05-08

**Authors:** Ana Onu, Daniela-Marilena Trofin, Andrei Tutu, Ilie Onu, Anca-Irina Galaction, Dragos-Petrica Sardaru, Dan Trofin, Cristiana Amalia Onita, Daniel-Andrei Iordan, Daniela-Viorelia Matei

**Affiliations:** 1 Doctoral School, “Grigore T. Popa” University of Medicine and Pharmacy Iasi, 700454 Iasi, Romania; chirila.ana@d.umfiasi.ro (A.O.); dr.danielatrofin@gmail.com (D.-M.T.); andrei.tutu@umfiasi.ro (A.T.); 2 Department of Biomedical Sciences, Faculty of Medical Bioengineering, “Grigore T. Popa” University of Medicine and Pharmacy Iasi, 700588 Iasi, Romania; dragos-petrica.sardaru@umfiasi.ro (D.-P.S.); trofin.dan@umfiasi.ro (D.T.); cristiana-amalia.onita@umfiasi.ro (C.A.O.); daniela.matei@umfiasi.ro (D.-V.M.); 3 Department of Morpho-Functional Sciences II (Pathophysiology), Center for Obesity BioBehavioral Experimental Research, ”Grigore T. Popa” University of Medicine and Pharmacy Iasi, 700115 Iasi, Romania; 4 Department of Individual Sports and Kinetotherapy, Faculty of Physical Education and Sport, “Dunarea de Jos” University of Galati, 800008 Galati, Romania; daniel.iordan@ugal.ro; 5 Center of Physical Therapy and Rehabilitation, “Dunărea de Jos” University of Galati, 800008 Galati, Romania

**Keywords:** metabolic syndrome, oxidative stress, physical exercise, dietary strategies, ketogenic diet, Mediterranean diet, dietary approaches to stop hypertension, intermittent fasting, inflammation

## Abstract

Metabolic syndrome (MetS) is characterized by central obesity, insulin resistance, hypertension, dyslipidemia, and chronic inflammation, significantly increasing the risk of cardiovascular disease and type 2 diabetes. Effective management of MetS is critical, with exercise being a key intervention. This review analyzed the effects of different exercise intensities—low, moderate, and high-intensity interval training (HIIT)—on metabolic health, oxidative stress (OS), inflammation, and cardiovascular function. A search of Medline, PEDro, and EBSCO identified 2251 articles, with 159 studies published between 1999 and 2025 included after screening. Low-intensity exercise improved insulin sensitivity, reduced OS markers (e.g., MDA, 8-OHdG), and enhanced antioxidant enzyme activity. Moderate-intensity exercise showed similar benefits with notable reductions in inflammatory markers (e.g., IL-1β, TNF-α). HIIT promoted fat loss and improved metabolic markers but temporarily increased OS and inflammation. Dietary strategies also play a critical role. The Mediterranean diet and Dietary Approaches to Stop Hypertension (DASH) diets are well established, emphasizing nutrient-dense foods like unsaturated fats and fiber to reduce inflammation and manage weight. The ketogenic diet (KD), a high-fat, low-carbohydrate approach, has recently gained attention for its metabolic benefits. KD induces ketosis, improving insulin sensitivity, reducing triglycerides, and enhancing fat oxidation. Studies show KD effectively reduces body weight and glucose levels, though long-term adherence and nutrient deficiencies remain challenges. Intermittent fasting also showed potential benefits, though effects on glucose metabolism were inconsistent. This review underscores the need for tailored approaches combining exercise, diet, and fasting to optimize MetS outcomes, offering integrative strategies for prevention and management.

## 1. Introduction

Metabolic syndrome (MetS) is a group of conditions that together increase the risk of cardiovascular disease, diabetes mellitus type 2 (DM2), stroke, and atherosclerosis, and it has become a significant global health concern.

The World Health Organization (WHO) definition, established in 1999, requires the presence of insulin resistance or elevated blood glucose levels [> 6.1 mmol/L (110 mg/dL)], along with two or more of the following: low high-density lipoprotein cholesterol (HDL-c) [<0.9 mmol/L (35 mg/dL) in men, <1.0 mmol/L (40 mg/dL) in women], high triglycerides [>1.7 mmol/L (150 mg/dL)], a BMI > 30 kg/m^2^, or high blood pressure [>140/90 mmHg] [1].

MetS, also known as syndrome X or insulin resistance, is a pathological condition characterized by abdominal obesity, insulin resistance, hypertension, and hyperlipidemia. Its prevalence has risen globally due to increased consumption of high-calorie, low-fiber foods and decreased physical activity linked to urbanization and sedentary lifestyles. This syndrome significantly contributes to DM2, cardiovascular diseases, and stroke, imposing a massive economic burden on healthcare systems. Effective prevention strategies include urban planning that promotes physical activity, policies that encourage healthier food choices, and restrictions on advertising unhealthy foods. Without large-scale societal interventions, the current trend is unsustainable, highlighting the urgent need for lifestyle modifications to combat this growing health crisis [2].

The definitions of MetS have evolved in time to improve diagnostic accuracy and applicability. The National Cholesterol Education Program Adult Treatment Panel III (NCEP: ATP III) (2004) allowed diagnosis based on any three out of the five components, with relatively high waist circumference thresholds. In contrast, the International Diabetes Federation (IDF) (2005) made central obesity a mandatory component and lowered waist circumference cut-offs for the Caucasian population [3]. The IDF Consortium (2009) retained the option of using BMI ≥ 30 kg/m^2^ as an alternative to waist circumference. The 2022 Polish Experts Consensus maintained the mandatory obesity criterion and also accepted BMI ≥ 30 kg/m^2^. Blood pressure thresholds remained mostly consistent, but from 2005 onward, home measurements were also considered valid [3]. Hyperglycemia definitions became more comprehensive, including fasting glucose, 2 h oral glucose tolerance test results, and HbA1c values. Dyslipidemia criteria expanded in 2022 to include non-HDL-c instead of only HDL-c and triglycerides [3].

## 2. State-of-the-Art

MetS is represented by an accumulation of metabolic abnormalities such as insulin resistance, hypertension, central obesity, high levels of low-density lipoprotein (LDL)-cholesterol, and low levels of HDL cholesterol. These factors significantly increase the risk of developing serious complications such as DM2 and atherosclerotic cardiovascular disease [4].

Numerous studies show links between chronic stress and MetS [5,6,7]. The pathophysiology induced by chronic stress is mainly attributed to the sympathetic nervous system (SNS) axis and the hypothalamic–pituitary–adrenal (HPA) axis [8,9]. Along with these axes, the Paraventricular nucleus–sympathetic–adipose pathway axis and the microbiota-gut-brain axis are also involved in MetS.

### 2.1. Autonomic Nervous System and Hypothalamic–Pituitary–Adrenal Axis Dysregulations in MetS

The ANS plays a major role in feeding behavior, fuel mobilization, energy utilization, and energy storage [10]. The key energy-sensing regions of the brain, such as the ventromedial hypothalamic nucleus (VMH), arcuate nucleus (ARC), dorsomedial hypothalamic nucleus (DMH), and the paraventricular nucleus (PVN), detect and regulate endocrine factors, such as insulin, ghrelin, and leptin [11,12,13].

Stress-induced elevations in glucocorticoids are associated with profound metabolic abnormalities, including insulin resistance, glucose intolerance, dyslipidemia, increased central adiposity, and hypertension, suggesting that chronic stress may in part contribute to the development of the MetS [14].

After a meal, the normal physiological response in healthy humans is to increase sympathetic nerve activity. Increased central sympathetic activity in the postprandial state is important to promote thermogenesis and to produce peripheral vasoconstriction to maintain blood pressure following splanchnic vasodilatation [15].

Chronic increases in sympathetic nerve activity and a decrease in parasympathetic activity contribute to weight gain [16]. Chronic sympathetic overactivity in obesity is an adaptive physiological response used to stimulate thermogenesis and stabilize body weight during periods of overeating [17]. In MetS, an unbalance sympathovagal with sympathetic dominance and a lower vagal tone was demonstrated [18].

Increased SNS, observed in obese individuals, increases vascular constriction and impairs glucose transportation into the cells; these vascular constrictions have been implicated in insulin resistance [19]. Also, increased SNS has been demonstrated in obese patients who can produce selective leptin resistance, hyperinsulinemia, and low ghrelin levels [20,21]. Research highlights that SNS-mediated energy expenditure via brown adipose tissue (BAT) thermogenesis is significantly diminished in obesity. Studies also show that impaired parasympathetic activity indirectly affects BAT and gut function, contributing to disrupted energy homeostasis [13,14,22]. The effect of the parasympathetic nervous system (PNS) on BAT appears to be indirect by mediating the peripheral action of ghrelin, thus inhibiting sympathetic traffic directed to BAT [22]

### 2.2. The Role of Adipocytokines in Hypertension and MetS

#### Paraventricular Nucleus–Sympathetic–Adipose Pathway Axis

Adipose tissue, particularly in central obesity, functions as an endocrine organ, secreting adipocytokines that regulate metabolic and vascular functions. Key adipocytokines such as leptin, TNF-α, IL-6, and angiotensinogen contribute to hypertension by stimulating sympathetic SNS activity and impairing endothelial function [23,24].

White adipose tissue (WAT) is a major component of the body’s adipose tissue and is a source of fatty acids that are used in oxidative phosphorylation of adenosine triphosphate (ATP) and generate energy for body requirements [25]. WAT, receiving sympathetic nerve intervention, is not only the major site of energy storage but also an active endocrine organ. Increased sympathetic activity stimulates adipocyte lipolysis by binding to beta-adrenergic receptors in WAT to activate cAMP-dependent pathways to translocate inactive lipase [25]. In a recent study, authors found that the Paraventricular nucleus –sympathetic nerve–adipose circuit is important for the regulation of adipokine expression and subsequent insulin resistance and depressive-like behaviors induced by chronic restraint stress [26]. WAT also controls appetite, intervenes in the metabolism of carbohydrates and fat, and regulates thermogenesis and immunity [27,28].

Adiponectin is a protein hormone. Adiponectin improves hepatic insulin resistance by reducing glycogenesis and lipogenesis and increasing glucose utilization [29] and also improves vascular function by stimulating endothelial NO production and antiatherogenic effects by inhibiting inflammation in vascular systems [30]. A high-fat diet causes adiponectin resistance in skeletal muscle and liver tissue, causing the development of systemic hyperglycemia and hyperlipidemia [31]. However, a diet high in polyunsaturated fatty acids (PUFAs), especially omega-3, increases the gene expression and plasma level of adiponectin. Adiponectin has emerged as a potential therapy for DM2, as it can increase the insulin sensitivity of the liver and skeletal muscle [32].

Leptin, a key regulator of appetite and energy expenditure, influences vasoconstriction and SNS activation, linking obesity to hypertension. Chronic hyperleptinemia, often observed in obesity, results in “selective leptin resistance”, where its appetite-suppressing effects wane while its vascular effects persist. This imbalance exacerbates hypertension [33,34]. The distinct roles of adipocytokines like leptin and adiponectin in MetS demonstrate their potential as biomarkers and therapeutic targets. Modulating their expression and activity through pharmacological or dietary means could alleviate MetS-associated hypertension and systemic inflammation.

### 2.3. Oxidative Stress and Inflammation in MetS

Oxidative stress (OS) emerges as a central driver of MetS, characterized by an imbalance between ROS production and antioxidant defenses. Excess ROS damages cellular structures, disrupts mitochondrial function, and impairs nitric oxide bioavailability, thereby worsening endothelial dysfunction and hypertension [35]. Savaş et al. 2020 demonstrated the importance of oxidative stress and inflammation in MetS by investigating the roles of macrophage apoptosis inhibitor (AIM) and monocyte chemotactic protein-1 (MCP-1). Their study revealed that individuals with MetS exhibited significantly elevated serum levels of AIM, MCP-1, and C-reactive protein (CRP), correlating strongly with waist circumference. AIM and CRP were identified as independent predictors of MetS, while MCP-1, although not independent, highlighted the contribution of inflammation to MetS pathology. Furthermore, the migration of M1 macrophages to visceral adipose tissue was identified as a key process linked to oxidative stress [36].

Monserrat-Mesquida et al. 2020 conducted a study on 160 adults aged 55 to 80 years in the Balearic Islands, identifying elevated levels of plasma malondialdehyde (MDA), a marker of OS, and pro-inflammatory cytokines such as TNF-α and IL-6 in individuals with MetS. Concurrently, antioxidant enzyme activity, including superoxide dismutase (SOD), was reduced. Interestingly, men with MetS demonstrated higher myeloperoxidase activity, suggesting sex-specific differences in oxidative stress responses. Increased catalase (CAT) and glutathione reductase (GRd) activities in peripheral blood mononuclear cells (PBMCs) indicated a compensatory response to oxidative stress, likely driven by preactivated immune cells [37]. These studies illustrate the interconnected roles of OS and inflammation in MetS progression. Targeting inflammatory pathways and enhancing antioxidant defenses represent promising strategies for mitigating MetS-related complications.

Inflammation and OS are interconnected, creating a vicious cycle in which inflammatory cytokines induce ROS production, and ROS, in turn, amplify inflammatory pathways.

### 2.4. Hypertension and Its Links to Insulin Resistance

There is a clear link between hypertension, insulin resistance, hyperglycemia, and hyperinsulinemia. Hyperinsulinemia is related to increased plasma catecholamine levels, regardless of blood glucose levels. In addition, hypertension may result from alterations in sodium and water metabolism, in particular through insulin-fostered reabsorption in the proximal renal tubules [38,39]. Experimental studies have shown that animal hypertension can be induced by feeding diets rich in fructose and sucrose, which contribute to increased sympathetic activity [39]. These findings suggest that dietary factors such as high fructose or sucrose intake may increase blood pressure by promoting insulin resistance and hyperinsulinemia. In addition, insulin resistance is associated with increased levels of free fatty acids, which can further exacerbate hypertension. Free fatty acids promote vasoconstriction by generating ROS species, which damage blood vessels and contribute to increased blood pressure [40]. Insulin resistance exacerbates hypertension through increased sodium reabsorption, loss of vasodilation, vasoconstriction caused by free fatty acids, and sympathetic hyperactivation.

### 2.5. Genetic Variations in Metabolic Syndrome

MetS and its components remain complex and incompletely understood, complicating the identification of critical clinical determinants. Both genetic and environmental factors contribute significantly to MetS development. Numerous studies have explored the association between genes, polymorphic variants, and MetS or its risk factors. Research across diverse populations underscores the widespread relevance of metabolic abnormalities regardless of age or genetic background. Key polymorphisms identified include IL-6 (rs1800796), HSPA1B (1267 A > G), and IL-18 (rs187238, rs1946518). Additional associations involve ABCG1, TNF-α (rs1800629), CYP2R1, VDR, and DRD2 genes. Significant correlations have also been reported with variants in VEGF, SREBF2 (rs1052717, rs4822064), TRPM5 (rs4929982), and FTO (rs9939609). Other implicated genes include eNOS, PAI-1, ACE, OXTR, CAT-21A/T, ATR1 A1166C, and SOD1 (35 A > C). Polymorphisms in ADIPOQ (rs2241766, rs1501299), GSTT1, GSTM1, and GSTP1 further highlight the genetic complexity of MetS [41,42,43,44,45,46,47,48,49].

Kochetova et al. 2022 investigated the association of polymorphic variants in inflammation-related genes (CRP, TNFA, TNFRSF1B) with MetS and serum levels of TNF-α and hsCRP. In a case-control study, 271 MetS patients and 327 healthy controls were genotyped for specific SNPs. The TNFRSF1B (rs1061624) variant was associated with MetS and TNF-α levels, suggesting a potential protective effect of the AA genotype in this population. The TNFA (rs1800629) SNP was linked to higher TNF-α levels and albuminuria. CRP (rs2794521) was associated with insulin resistance, hsCRP, waist-hip ratio, and BMI, with certain haplotypes showing a stronger association with MetS markers. These findings confirm that CRP, TNFA, and TNFRSF1B genes are involved in the pathogenesis of MetS. Specifically, the TNFRSF1B gene variant rs1061624 has not been previously associated with MetS, marking a novel discovery. CRP gene polymorphisms also contribute to the inflammatory response in MetS patients, influencing key metabolic traits [50].

A recent study by Masood et al. explores the association between genetic variants of visfatin and MetS. Visfatin, also known as pre-B cell colony-enhancing factor (PBEF) or nicotinamide phosphoribosyltransferase (NAMPT), is an adipokine involved in glucose homeostasis, lipid metabolism, and inflammation. The genotypic frequencies of visfatin SNPs, including rs2302559 (OD: 18.222; 95% CI 10.228–32.466; *p*-value < 0.001) and rs1215113036 (OD: 129.40; 95% CI 44.576–375.693; *p*-value < 0.001), were significantly associated with MetS. Mutant alleles of both SNPs were more frequent in patients with MetS compared to the control group. These findings suggest that genetic variations in visfatin, such as rs2302559 and rs1215113036, increase the likelihood of developing MetS [51].

This review offers valuable insights into how different exercise intensities affect metabolic and cardiovascular health, particularly in the context of MetS. It compares the effects of low-intensity, moderate-intensity, and high-intensity interval training (HIIT), highlighting how each exercise type influences metabolic parameters, OS, inflammation, and cardiovascular function. The review clarifies both the benefits and potential risks associated with each intensity level, providing guidance tailored to individuals with MetS.

Furthermore, it emphasizes the role of exercise in modulating the autonomic nervous system (ANS), including sympathetic (SNS) and parasympathetic (PNS) activity, as well as the HPA axis—critical components of MetS pathophysiology that remain underexplored in prior studies.

In addition, the review incorporates dietary interventions such as the Mediterranean diet, the Dietary Approaches to Stop Hypertension (DASH) diet, the ketogenic diet, and intermittent fasting. These are examined for their capacity to support metabolic and cardiovascular health, offering a comprehensive and synergistic strategy for MetS management.

Importantly, the review also addresses the potential risks of HIIT, such as elevated OS and inflammation, and recommends a cautious, individualized approach, especially for individuals with cardiovascular conditions or hypertension.

Ultimately, the review advocates for a personalized treatment strategy for MetS, accounting for the varying effects of different exercise intensities and dietary patterns on inflammation, oxidative stress, and metabolic function. This work contributes to the development of tailored exercise and nutrition programs aimed at improving metabolic health and preventing complications associated with MetS.

## 3. Methods

For this review, we conducted a search of open-access articles related to MetS, OS, antioxidant defense mechanisms, chronic diseases, cardiovascular health, and lifestyle interventions, including both physical exercise and dietary strategies. Our focus extended to studies exploring the effects of various exercise intensities (low, moderate, and high) and dietary approaches such as the Mediterranean diet, ketogenic diet, intermittent fasting, and the DASH (Dietary Approaches to Stop Hypertension) diet.

We considered the impact of these interventions on key health parameters, including blood glucose levels, lipid profiles, inflammation, and OS markers. We also included articles that examined underlying mechanisms of action, physiological benefits, and the effects of interventions on both physical and biochemical markers in individuals with conditions such as MetS, hypertension, and DM2.

Searches were conducted across major international databases: Medline, PEDro, and EBSCO. A total of 159 relevant articles published between 1999 and 2025 were selected. This review details each step of the selection and screening process from initial identification to final inclusion. This includes the number of records identified through database searches, duplicates removed, records screened, full-text articles assessed for eligibility, and studies ultimately included.

The inclusion criteria were strictly defined to ensure relevance and methodological quality. Articles were eligible if they contained key terms in the title, abstract, or keywords. Included study types were: cross-sectional studies, non-randomized controlled trials, randomized controlled trials, observational studies, and review articles. Studies had to examine either exercise interventions, dietary strategies (including Mediterranean, ketogenic, DASH diets, and intermittent fasting), or a combination thereof, in relation to MetS and related markers.

To ensure methodological consistency, only articles meeting predefined eligibility criteria were included. Studies had to feature relevant keyword combinations in their titles or abstracts. Eligible study types included cross-sectional studies, non-randomized controlled trials, randomized controlled trials, observational studies, and review articles.

Exclusion criteria were studies with fewer than sixteen participants, duplicate publications or overlapping datasets, and articles deemed irrelevant based on the predefined inclusion criteria.

A total of 2251 articles were identified through searches of Medline (906 articles), Pedro (87 articles), and EBSCO (1258 articles) databases, using the following search strategy: “metabolic syndrome” (MeSH Terms) OR [“metabolic” (All Fields) AND “syndrome” (All Fields)] “oxidative stress” (MeSH Terms) OR [“oxidative” (All Fields) AND “stress” (All Fields)] “inflammation” (MeSH Terms) OR [“inflammation” (All Fields)] “physical exercise” (MeSH Terms) OR [“physical” (All Fields) AND “exercise” (All Fields)] “low-intensity exercise” (MeSH Terms) OR [“low-intensity” (All Fields) AND“exercise” (All Fields)] “moderate-intensity exercise” (MeSH Terms) OR [“moderate-intensity” (All Fields) AND “exercise” (All Fields)] “high-intensity interval training” (MeSH Terms) OR [“high-intensity” (All Fields) AND “interval” (All Fields), AND “training” (All Fields)] “dietary intervention” (MeSH Terms) OR Terms) OR [“dietary” (All Fields) AND AND “intervention” (All Fields)] “mediterranean diet” (MeSH Terms) OR [“mediterranean” (All Fields) AND “diet” (All Fields)] “dietary approaches to stop hypertension” (MeSH Terms) OR [“dietary” (All Fields) AND “approache” (All Fields) AND “to” (All Fields) AND “stop” (All Fields) AND “hypertension” (All Fields)] “ketogenic diet“ (MeSH Terms) OR [“ketogenic” (All Fields) AND “diet” (All Fields)] “intermittent fasting (MeSH Terms) OR [“intermittent” (All Fields) AND “fasting” (All Fields)] After initial screening, 1358 articles were excluded based on title and abstract review. The remaining 1385 full-text articles were evaluated for eligibility. Following further evaluation, 734 additional articles were excluded because of a focus on conditions outside the specified domains and keywords.

## 4. Results

### 4.1. Physical Exercise

Systematic physical exercise is a key intervention for modifying the clinical components of MetS, as it leads to improvements in insulin sensitivity, lipid profiles, blood pressure, and body composition. Exercise refers to any structured movement that requires energy and is repetitive, planned, and purposeful. Regular moderate physical exercise has been shown to improve these key metabolic parameters and reduce risk factors associated with MetS [8,51,52,53,54]. Low levels of physical fitness are considered significant risk factors for the development of Mets and overall mortality, highlighting the importance of maintaining an active lifestyle for improving health outcomes.

Gleeson et al. demonstrated that regular exercise has significant anti-inflammatory effects, largely by reducing visceral fat mass. Visceral fat is often associated with increased production of proinflammatory adipokines, which contribute to the chronic low-grade inflammation seen in conditions such as MetS. In addition, the anti-inflammatory effects of exercise may also occur independently of changes in fat mass, suggesting that the physiological benefits of exercise extend beyond simple fat reduction [55].

Allen et al. 2015 demonstrated that exercise induces a cascade of inflammatory responses. During and after exercise, skeletal muscle releases myokines and cytokines that help regulate metabolic and inflammatory processes. Regular moderate exercise reduces systemic inflammation, evidenced by a decrease in acute phase proteins. Exercise increases anti-inflammatory cytokines such as IL-1RA and IL-10 while reducing pro-inflammatory cytokines such as IL-1β and TNF-α [56].

Nishii et al. 2023 showed that exercise promotes the release of myokines, such as IL-6 and other peptides, produced by muscle fibers, which help protect against inflammation-related diseases. The anti-inflammatory effects of regular exercise are due in part to the modulation of intracellular signaling pathways, particularly those mediated by NO and oxygen free radicals. Increased production of NO and oxygen-free radicals during exercise is essential for triggering anti-inflammatory defense mechanisms in the body. The benefits of exercise on inflammation extend beyond muscular activity, involving broader metabolic and immune system responses [57].

Scheele et al. demonstrated that exercise temporarily increases the production of ROS and inflammatory cytokines in skeletal muscle. Although ROS were traditionally considered harmful to cells, recent research has highlighted their role in regulating cell signaling, promoting cytokine production, and supporting muscle adaptation to exercise. Myokines are distinct from typical pro-inflammatory cytokines as they offer anti-inflammatory and metabolic benefits. On the other hand, excessive ROS, when not adequately neutralized by the body’s defenses, can lead to OS, a condition characterized by the accumulation of free radicals that cause cellular damage. The dual nature of ROS—acting both beneficially and harmfully—emphasizes the complexity of their role in muscle function and overall health [58].

In 2024, Monserrat-Mesquida et al. investigated the impact of regular physical activity on OS in older adults with MetS. Results indicate that higher levels of physical activity were associated with a significant reduction in OS markers, particularly 8-dihydroguanosine (8-OxoGuo), a marker of nucleoside damage. This suggests that exercise may reduce oxidative damage to RNA, which is linked to a reduced risk of cardiovascular disease and insulin resistance. In addition, participants with higher levels of physical activity experienced reductions in proinflammatory cytokines such as IL-6 and IL-1β, which are often elevated in OS states. These findings highlight the interconnection between OS and inflammation, where exercise has dual benefits by attenuating both. Physical activity also improved glycemic control, with participants exhibiting lower fasting glucose and glycosylated hemoglobin levels (HbA1c), further supporting the role of exercise in the management of MetS [59].

#### 4.1.1. Low-Intensity Exercise

**Figure 1:**
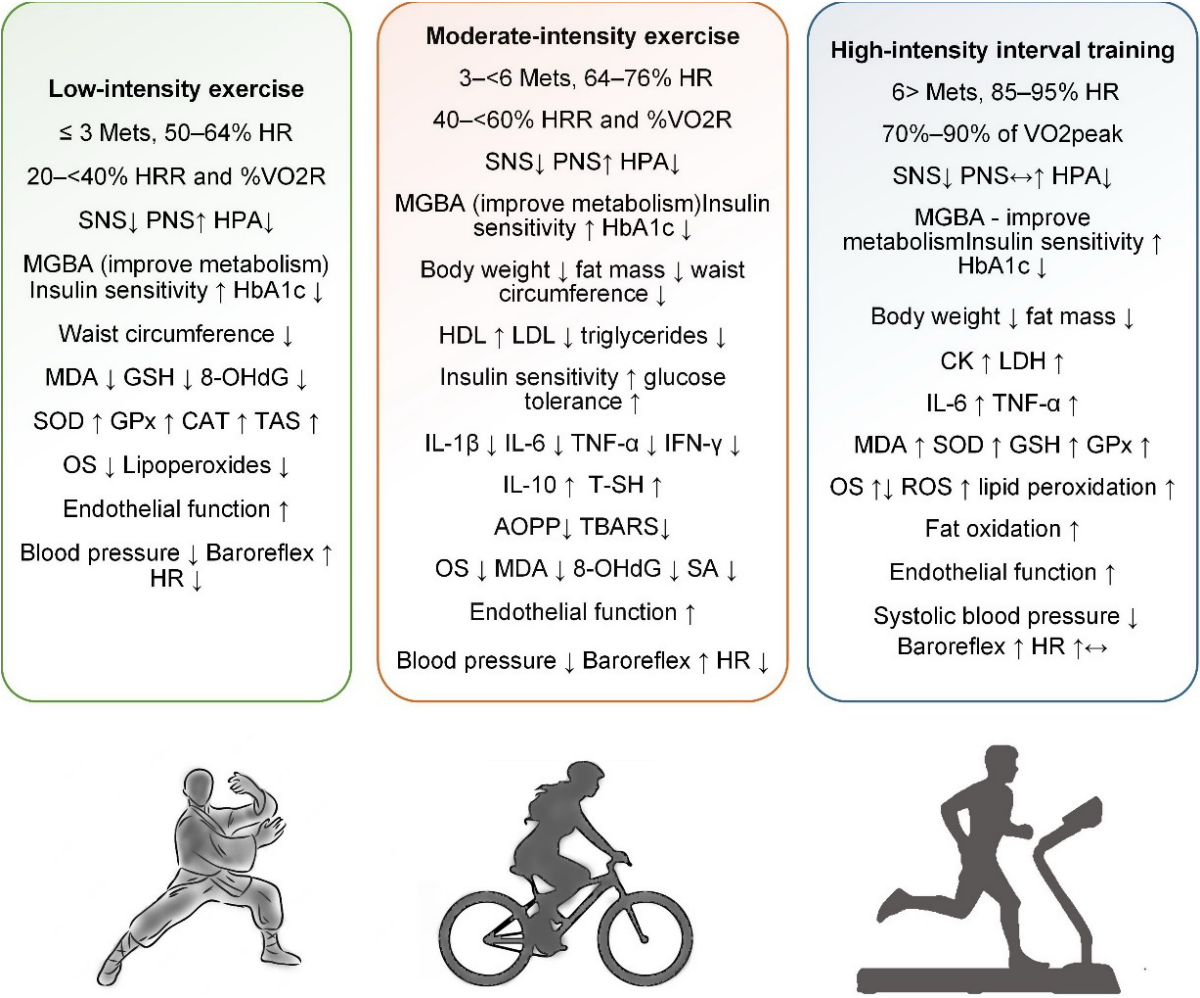
Physical exercise in metabolic syndrome in terms of effects on the sympathetic and parasympathetic nervous system, metabolism, oxidative stress, anti- and pro-inflammatory effects, cardiac activity, glycemic control, body weight, and adipose tissue (increase↑, decrease↓).

Low-intensity exercise was defined as an activity performed at 20 < 40% heart rate reserve (HRR) and oxygen uptake reserve (%VO2R), corresponding to 50–64% of maximum heart rate and energy expenditure of less than three metabolic equivalent tasks (Figure 1) [60]. Low-intensity exercises, such as walking, yoga, and tai chi, are increasingly recognized for their significant health benefits, particularly for people with chronic conditions or those new to exercise. These activities prioritize safety while strengthening cardiovascular health, improving mobility, and reducing stress. In addition, they support long-term adherence to fitness regimes due to their sustainable nature and reduced physical demands. Evidence from clinical trials suggests that even moderate activity levels, such as walking 30 min a day, can significantly reduce the risk of chronic diseases, including hypertension and diabetes [61].

Cramer et al. 2016 conducted a systematic review and meta-analysis to assess yoga’s impact on its components. A comprehensive search of multiple databases, including MEDLINE/PubMed, Scopus, and the Cochrane Central Register of Controlled Trials, identified seven randomized controlled trials involving 794 participants. The results showed no significant effects of yoga on the complete resolution of MetS or key parameters such as triglycerides, HDL-c, and fasting plasma glucose. However, yoga showed a small but significant reduction in waist circumference and systolic blood pressure (SBP) compared with usual care. Specifically, yoga reduced waist circumference (SMD = −0.35, 95% CI = −0.57 to −0.13; *p* < 0.01) and SBP (SMD = −0.29, 95% CI = −0.51 to −0.07; *p* <0.01). However, these findings were not robust against selection bias [62].

Another systematic review by Khoshnaw and Ghadge investigated the effects of yoga on the main risk factors for MetS, including glucose homeostasis, lipid profile, adipocytokines, and cardiovascular health, and explored possible mechanisms by which yoga may exert its effects. A search of MEDLINE/PubMed, Scopus, and the Cochrane Library yielded mixed results on the impact of yoga on MetS risk factors. Although some studies suggest beneficial effects, particularly in improving insulin sensitivity and reducing blood pressure, the evidence remains inconsistent and insufficient to definitively recommend yoga as an effective intervention for MetS [63].

Patil et al. 2014 conducted a study that aimed to investigate the effect of yoga on OS markers in elderly individuals with grade I hypertension. A randomized controlled trial was conducted with 57 elderly male participants (aged 60–80 years) with grade I hypertension. The participants were divided into a yoga group (intervention) and a control group that performed walking exercises for one hour a day, six days a week for three months. Results showed that the yoga intervention significantly reduced serum MDA levels (*p* < 0.001) while improving antioxidant levels, including serum SOD activity (*p* < 0.007), reduced glutathione (GSH) (*p* < 0.002), and vitamin C levels (*p* < 0.002). In contrast, the control group had a significant increase in serum MDA levels (*p* < 0.04) and a reduction in vitamin C levels (*p* < 0.015), with no significant changes in SOD and GSH levels. These results suggest that yoga is an effective intervention for reducing OS and improving antioxidant defense mechanisms in elderly hypertensive individuals [64].

Venugopal et al. 2022 in a systematic review and meta-analysis, evaluated the effect of yoga on OS in adults with DM2. OS is associated with free radicals, and decreased antioxidant defense is a key factor in the development of DM2 complications. The main focus was on MDA, and secondary outcomes such as fasting plasma glucose, HbA1c, and SOD levels. Results indicated that yoga significantly reduced MDA levels, suggesting a decrease in OS (SMD: −1.4, *p* < 0.03). Yoga also reduced fasting plasma glucose (SMD: −1.87, *p* = 0.06) and HbA1c (SMD: −1.92, *p* < 0.0007), demonstrating improvements in glycemic control. However, no significant effect was observed on SOD levels (SMD: −1.01, *p* = 0.56) [65]. The results support yoga as an adjunctive therapy to reduce OS and improve glycemic control in patients with DM2.

Promsrisuk et al. 2023 conducted a study that investigated the effects of combining elastic band resistance exercise (EBRE) with modified Thai yoga on blood glucose, OS, antioxidants, lung function, respiratory muscle strength, and airway inflammation in elderly patients with DM2. Forty-two patients with DM2 were randomly assigned to either an exercise group (*n* = 21) or a control group (*n* = 21) with a regular daily routine. The exercise group participated in EBRE combined with modified Thai yoga for 40 min, 5 days a week for 12 weeks. The results showed significant improvements in the exercise group: fasting blood glucose and HbA1c levels were reduced, and markers of OS, such as MDA, were significantly decreased. At the same time, antioxidants such as SOD and CAT were significantly increased. Lung function, as measured by forced expiratory volume (FEV1), forced vital capacity (FVC), and peak expiratory flow (PEF), showed significant improvement. In addition, combined exercise decreased airway inflammation, indicated by a reduction in Fractional exhaled Nitric Oxide (FeNO) [66].

In 2021, Rosado-Pérez et al. conducted a systematic review and meta-analysis to evaluate the effects of Tai Chi on OS markers, comparing it with sedentary behavior, walking, and yoga. Databases, such as MEDLINE, Cochrane Library, and ScienceDirect, were searched for randomized controlled trials (RCTs) and non-randomized controlled trials (NRCTs). A total of ten studies (five RCTs and five NRCTs) were included, which looked at outcomes such as SOD, CAT and lipoperoxides. Regular practice of Tai Chi significantly increased SOD (MD = 34.97 U/mL) and CAT (MD = 15.63 U/mL) while reducing lipoperoxides (MD = −0.02 µmol/L) compared to sedentary behavior [67]. However, only a single study has compared Tai Chi with walking or yoga, limiting conclusions regarding these comparisons. The findings suggest that Tai Chi improves antioxidant activity and reduces OS.

Mendoza-Núñez et al. 2018 investigated the impact of Tai Chi exercise on OS and inflammatory markers in older adults diagnosed with MetS. Conducted as a quasi-experimental study, it included 110 sedentary participants divided into a control group (*n* = 50) and an experimental group (*n* = 60) engaged in supervised Tai Chi training for 50 min per day, 5 days per week, for 6 months. The Tai Chi group showed significant improvements, including a reduction in HbA1c, suggesting hypoglycemic benefits, and an increase in total antioxidant status (TAS), along with a reduction in OS score (*p* < 0.05). However, no notable changes in cardiovascular parameters, such as blood pressure or heart rate, were observed [68].

In a recent study, Chang and Liu explored the impact of resistance training, Tai Chi, and their combination on OS, glycemia, and lipid metabolism in elderly patients with DM2. Ninety-four patients were randomly assigned to one of four groups: resistance training, Tai Chi, combined intervention, and a control group, with the exercise groups participating in a 24-week program. All participants received standard nutrition and medication. The interventions significantly reduced OS markers such as MDA and improved antioxidant enzyme levels, including SOD. Glycemic markers, such as fasting plasma glucose (FPG), postprandial plasma glucose (PPG), and HbA1c, also improved in the intervention groups. The combined Tai Chi and resistance training intervention showed the greatest reduction in OS markers, including 8-hydroxy-2′-deoxyguanosine (8-OHdG), indicating superior antioxidative effects [69]. However, the combined intervention did not provide additional benefits in terms of glycemic or lipid control compared with individual interventions.

Rosado-Pérez et al., in an original study, investigated the antioxidant effects of Tai Chi compared with walking in 106 clinically healthy older adults aged 60–74 years. The participants were divided into three groups: control (no intervention), walking, and Tai Chi, and OS markers were measured before and after the interventions. These markers included lipoperoxides, antioxidant enzymes [SOD and glutathione peroxidase (GPx)], and TAS. Post-intervention analysis showed that Tai Chi significantly reduced lipoperoxide levels compared to walking and the control group. In addition, Tai Chi improved SOD activity and reduced overall OS score more effectively than walking. The findings suggest that Tai Chi generates a stronger antioxidant response, potentially linked to delaying aging processes, compared to walking [70].

Low-intensity exercise, defined as activity performed at 20 < 40% HRR or <3 METs, includes practices like walking, yoga, and tai chi, which offer safe and sustainable health benefits. These exercises are particularly beneficial for individuals with chronic conditions or those beginning physical activity programs. Evidence suggests that regular low-intensity exercise can reduce the risk of chronic diseases such as MetS, hypertension, and DM2. Several studies have examined the effects of yoga on components of MetS and OS, with mixed but promising results. While systematic reviews show limited impact of yoga on lipid profiles or glycemia, improvements in WC, BP, and TAS have been observed. Tai chi has demonstrated significant antioxidant effects, including increases in SOD and CAT and reductions in lipoperoxides and HbA1c. Combined interventions, such as resistance training with tai chi or yoga, appear to enhance antioxidant defenses and glycemic control. Yoga has also been shown to reduce serum MDA and improve antioxidant enzymes like SOD and GSH. However, evidence across studies remains inconsistent due to methodological variations (Table S1). Overall, low-intensity exercise shows potential as an adjunctive strategy for managing MetS and reducing OS, particularly in elderly and high-risk populations (Figure 1).

#### 4.1.2. Moderate-Intensity Exercise

Moderate-intensity exercise was defined as an activity performed at 40 < 60% HRR and %VO2R, corresponding to 64–76% of maximum HR and energy expenditure of three to six metabolic equivalent tasks (Figure 1) [60]. Examples include brisk walking, bicycling on flat terrain, playing doubles tennis, swimming, water aerobics, treadmill walking, or recreational volleyball and basketball. According to the American Heart Association Recommendations for Physical Activity in Adults and Kids, adults should perform at least 150 min of moderate activity per week, divided into sessions of at least 10 min, or the equivalent of intense activity (75 min per week). These moderate-intensity exercises, when performed regularly, have been shown to significantly reduce the risk of several chronic conditions, including heart disease, stroke, DM2, high blood pressure, and obesity [71].

Carroll and Dudfield emphasize the essential role of moderate-intensity physical training in the prevention and treatment of MetS. While moderate physical activity is widely recommended as part of “therapeutic lifestyle change”, evidence highlights the specific benefits of supervised, long-term, moderate to moderately vigorous physical training. These interventions improve the dyslipidemic profile by raising HDL-c and lowering triglycerides, even without therapeutic weight loss [72]. Combined with dietary modifications, exercise can increase insulin sensitivity, improve glucose tolerance, and effectively prevent or delay the onset of DM2 in people with impaired glucose regulation. In addition, exercise training reduces blood pressure in overweight and obese individuals with elevated blood pressure, further addressing MetS risk factors.

Tjønna et al. investigated the effects of continuous moderate exercise (CME, 70% of maximum heart rate) versus aerobic interval training (AIT, 90% of maximum heart rate) on cardiovascular function and prognosis in patients with MetS. Thirty-two patients participated in a 16-week program with equal exercise volumes between groups. AIT led to a greater increase in maximal VO_2_ (35% vs. 16%; *p* < 0.01) and a greater reduction in MetS risk factors compared with CME. AIT was also superior in improving endothelial function, insulin signaling, muscle biogenesis, and glucose regulation [73]. Both exercise regimens were equally effective in lowering blood pressure, reducing body weight, and decreasing fat mass.

Ostman et al. 2017 assessed the impact of exercise training on clinical outcomes in individuals with MetS, including studies from multiple databases, including MEDLINE and Cochrane, focusing on trials lasting 12 weeks or more. Sixteen studies with 23 intervention groups were analyzed, totaling 77,000 patient hours of exercise. The results showed significant reductions in body mass index (BMI) (mean difference [MD] −0.29 kg/m², 95% CI −0.44, −0.15, *p* < 0.0001), body mass (MD −1.16 kg, 95% CI −1.83, −0.48, *p* < 0.0008), and waist circumference (MD −1.37 cm, 95% CI −2.02, −0.71, *p* < 0.0001) for participants undergoing aerobic exercise compared to the control group. Additionally, peak VO_2_ (MD 3.00 mL/kg/min, 95% CI 1.92, 4.08, *p* < 0.000001), systolic and diastolic blood pressure, fasting blood glucose (MD −0.16 mmol/L, 95% CI −0.32, −0.01, *p* = 0.04), triglycerides (MD −0.21 mmol/L, 95% CI −0.29, −0.13, *p* < 0.00001), and LDL (MD −0.03 mmol/L, 95% CI −0.05, −0.00, *p* = 0.02) levels all improved with aerobic exercise [74]. When comparing combined exercise to the control, waist circumference, peak VO_2_, SBP, and HDL levels showed significant improvements. However, no substantial differences between the two exercise interventions were observed. Exercise training led to improvements in body composition, cardiovascular health, and metabolic markers in individuals with MetS [74]. While both aerobic and combined exercises yielded positive outcomes, some measures seemed to benefit more from aerobic exercise alone.

In an original study, Lwow et al. 2011 examined how moderate-intensity exercise affects OS in postmenopausal women with different obesity phenotypes: metabolically healthy obese (MHO) and non-metabolically healthy obese (non-MHO). A total of 161 women participated, divided into three groups: metabolically healthy nonobese (MH-NO), MHO, and non-MHO. Each group exercised for 30 min at 50% of their maximal oxygen consumption, and OS was assessed by measuring thiobarbituric acid reactive substances (TBARS) and serum antioxidant activity (SA). Results showed no significant difference in SA between the MH-NO and MHO groups, but the non-MHO group had significantly lower SA than both other groups. Insulin resistance was the only factor that correlated with SA [75]. After exercise, TBARS levels increased in all groups, with no significant differences in increases between groups. The study concluded that TAS was better in MHO compared to non-MHO individuals, but all obese women, regardless of phenotype, experienced similar OS after exercise. The study suggests that the assessment of homeostasis patterns should be considered when developing exercise plans for obese individuals [75].

Farinha et al. 2015 investigated the effects of 12 weeks of aerobic training on OS and inflammatory markers in women with MetS. Twenty-three untrained women (mean age 51.86 years, BMI 30.8 kg/m^2^) participated in treadmill training without dietary modifications. Biomarkers such as AOPP (advanced oxidation protein products), TBARS, T-SH (total thiol content), Nox (relationship between nitrite/nitrate), and inflammatory cytokines (IL-1β, IL-6, IL-10, TNF-α, and IFN-γ) were measured before and after the intervention. The results showed significant reductions in IL-1β, IL-6, TNF-α, IFN-γ, AOPP, and TBARS, along with increases in IL-10 and T-SH levels [76]. However, NOx concentrations remained unchanged, and mRNA expression of inflammatory markers performed in peripheral blood mononuclear cells did not differ significantly. This suggests that although aerobic exercise improves systemic OS and inflammation, it does not alter gene expression in immune cells [76]. The findings highlight the health benefits of aerobic exercise for reducing oxidative damage and inflammation but also suggest that greater weight loss may be required to influence gene expression.

Poblete Aro et al. 2015 compared the effects of high-intensity interval training (HIIT) and CME on oxidative stress levels in patients with DM2. OS, caused by an imbalance between ROS and antioxidant defenses, is associated with the development of DM2. In this study, no significant differences were found in SOD levels between the training groups. However, HIIT led to a significant reduction in MDA compared to the control group and baseline (*p* < 0.05) [77]. Additionally, HIIT resulted in an increase in GPx levels and NO concentrations compared to both the CME group and baseline (*p* < 0.05). Both training types improved lipid profiles and fitness, but HIIT was more effective at normalizing OS by reducing pro-oxidant markers and boosting antioxidants [77]. This suggests that HIIT may be a more beneficial exercise modality for reducing OS in DM2 patients.

In a recent study, Rytz et al. explore the effects of aerobic exercise on OS markers among older, sedentary adults and examine how factors like MetS, sex, aerobic capacity, age, and weight influence these biomarkers. Two hundred and six participants (66.8 ± 6.4 years, 104 women) from the Brain in Motion study completed a six-month aerobic exercise intervention. Blood samples were collected at three time points to measure markers of OS (AOPP, MDA, 3-nitrotyrosine [3-NT]) and AS (CAT, uric acid [UA], SOD, ferric-reducing antioxidant potential [FRAP]). AOPP levels significantly decreased after 6 months of exercise (*p* < 0.003), with no difference based on MetS status (*p* = 0.183) [78]. However, participants with MetS had higher levels of AOPP, MDA, and FRAP than those without MetS. Men had higher FRAP, catalase, and UA levels across the intervention (*p* < 0.05). The interaction between sex and MetS revealed that the effects of MetS on OS markers were sex-dependent [78]. These findings highlight the complex relationship between aerobic exercise, MetS, sex, and OS, suggesting that gender-specific approaches may be important in managing conditions linked to OS.

Nojima et al., in an original study, evaluated the effects of moderate-intensity aerobic training on OS in patients with DM2 over 12 months. The participants were divided into three groups: aerobic training combined with a fitness center (group A, n = 43), aerobic training alone (group B, n = 44), and a control group (group C, n = 16). Participants in groups A and B exercised at 50% of VO_2max_ for at least 30 min, three times a week. Urinary levels of 8-OHd were measured. After 12 months, groups A and B showed a significant reduction in 8-OHdG levels, while group C showed no change. In addition, improvements in glycemic control, as measured by serum glycated albumin levels, were observed in groups A and B after 6 and 12 months. There was a positive association between changes in 8-OHdG levels and glycated albumin levels [79]. The study concluded that moderate-intensity aerobic exercise training reduced OS and improved glycemic control in patients with DM2.

Dekleva, Lazic, and Arandjelovic 2017 conducted a review examining the role of OS in hypertension and how exercise influences oxidative balance in hypertensive individuals. Exercise, especially chronic and intensive interval training, has been shown to improve antioxidant defense and reduce disease severity, whereas acute exercise may induce OS transiently. Regular aerobic physical activity is one of the most effective non-pharmacological methods to prevent and manage hypertension. Chronic aerobic exercise also improves endothelial function and overall cardiovascular health, with training at 50–70% of VO_2 max_ leading to better redox states and increased aerobic capacity. However, the effects of HIIT on OS in hypertensive patients are still unclear, and further research is needed to understand how different types of exercise modulate ROS levels and antioxidant defense [80]. While excessive ROS during exercise may cause damage, a certain level of ROS production may stimulate antioxidant mechanisms and enhance future cardiovascular adaptation, offering potential protection against hypertension.

Moderate-intensity exercise, defined as activity performed at 40–60% HRR or 64–76% of maximum HR, has proven benefits for preventing and managing MetS (Figure 1). Activities such as brisk walking, cycling, and swimming enhance cardiovascular fitness and reduce risk factors like hypertension, obesity, and insulin resistance. Studies have shown that even without weight loss, moderate-intensity training improves lipid profiles and glucose metabolism. Comparisons between CME and HIIT suggest HIIT may yield greater improvements in VO_2_ max and endothelial function. However, aerobic training consistently lowers OS markers and inflammatory cytokines across various populations, including those with DM2 and MetS. Meta-analyses confirm that aerobic exercise reduces BMI, WC, and fasting glucose while increasing HDL and peak VO_2_. While some evidence supports HIIT for superior antioxidant effects, moderate exercise remains effective and more accessible for many populations. OS and antioxidant response to exercise also vary by sex, obesity phenotype, and the presence of MetS. Long-term moderate aerobic training has been linked to improved redox balance and reduced oxidative DNA damage. Overall, regular moderate-intensity exercise is a powerful non-pharmacological tool to counteract MetS, improve metabolic health, and reduce systemic oxidative stress (Table S2).

#### 4.1.3. High-Intensity Interval Training

HIIT consists of short, intense bursts of exercise performed at 70–90% of VO2peak or 85–95% of maximum HR, followed by periods of active rest (Figure 1) [60]. These intervals allow for brief but intense efforts that push the body close to its maximum exertion level, improving cardiovascular fitness and metabolic function. The alternating high-intensity work and recovery periods make HIIT an efficient workout for improving overall physical performance in a short amount of time [81]. This approach is unique in its ability to develop the aerobic and anaerobic systems.

By pushing the body into the ‘red zone’ (exertion levels above 90% of VO_2 max_ and maximal HR), HIIT provides a powerful stimulus for significant cardiovascular and peripheral adaptations. Research conducted by Gibala and McGee in 2008 supports the idea that HIIT can be an effective alternative to traditional endurance training, often inducing similar or even superior improvements in physiological, performance, and health markers, applicable to healthy individuals and those with various conditions [82].

Recent studies have shown that HIIT can improve MetS in obese adults, optimize HbA1c in patients with DM2, and improve cardiac function in patients with myocardial infarction more than continuous moderate-intensity training [83,84].

Research conducted by de Araujo et al. in 2016 showed that HIIT can alter redox balance in both the short and long term, mainly due to the continuous production of ROS in the skeletal muscle bed. This increase in ROS can lead to elevated lipid peroxidation by disrupting the scavenging capacity of antioxidant enzymes [85]. The impact of exercise on OS appears complex and context-dependent, with the degree of OS being influenced by the specificity, load, intensity, and duration of training.

Interestingly, some evidence suggests that exercise may positively and negatively affect oxidative stress. For example, research conducted by de Sousa et al. 2016 found that regardless of intensity, volume, or type of exercise, there was a general trend of an increase in antioxidant indicators and a concomitant decrease in pro-oxidant indicators after exercise. This implies that exercise, including HIIT, could improve the body’s antioxidant defense mechanisms over time, potentially offsetting some of the negative effects of increased ROS production [86].

A recent study published in 2021 by Sarkar et al. showed that OS and inflammation increase with an 8-week HIIT program. This study recruited forty young male endurance athletes, who were divided into control and HIIT groups. The HIIT group underwent an 8-week sprint-HIIT program, involving three 3 h weekly sessions, consisting of four sprints at 90–95% maximal heart rate. Muscle damage (CK [creatine kinase] and LDH), inflammatory markers (IL-6, TNF-α), OS (MDA, SOD, GSH, and GPx), and physical fitness (VO_2max_) were assessed. Results showed significant increases in markers of muscle damage, inflammation, and oxidative stress, along with improvements in physical fitness parameters such as VO_2max_ and maximal strength [87]. However, elevated oxidative stress and inflammation suggest potential risks of overtraining and adverse health effects. The study highlights the need for HIIT to be supervised and tailored to individual athletes [87].

D’Alleva et al. 2022 investigated the effects of combined exercise training (COMB), a mixture of moderate-intensity continuous training (MICT) and HIIT, compared with HIIT alone on body composition, exercise capacity, and fat oxidation in obese adult males over 12 weeks. Thirty-four participants (mean age: 39.4 years; BMI: 34.0 kg/m^2^) were randomly assigned to the COMB (n = 18) or HIIT (n = 16) groups. Both groups completed ~36 training sessions with equivalent caloric expenditure. The COMB protocol included three repetitions of 2 min intervals at 95% VO_2_ peak, followed by 30 min at 60% VO_2_ peak, whereas the HIIT protocol involved 5–7 repetitions of 2 min intervals at 95% VO_2_ peak. Post-intervention, both groups showed significant reductions in body mass (mean: 3.09 kg) and fat mass (mean: 3.90 kg) [88]. VO_2_ peak improved similarly in both groups, with a mean of 0.47 L/min. The peak fat oxidation rate increased from 0.32 to 0.36 g/min in both groups. Additionally, HIIT sessions induced greater post-exercise fat oxidation due to elevated excess post-exercise oxygen consumption [88].

Li et al. compared the effects of HIIT and MICT training on blood pressure in patients with hypertension. A total of 13 randomized controlled trials involving 442 patients were included. The results showed no significant differences between HIIT and MICT in reducing resting SBP and diastolic blood pressure (DBP). However, HIIT was more effective than MICT in lowering SBP during daytime monitoring. HIIT also improved flow-mediated vasodilation more than MICT, indicating better vascular function. Subgroup analysis showed that HIIT was particularly effective in reducing SBP in hypertensive patients. Both HIIT and MICT were equally effective in reducing resting HR and improving VO_2_ max, with no significant differences between the two [89]. The study found that exercise intensity, rather than frequency or cycle length, may influence outcomes. HIIT produced greater shear stress on the vascular wall, which may explain its superior effect on flow-mediated vasodilation [89].

Romero-Vera et al., in a recent meta-analysis, assessed the effects of HIIT SBP and DBP in hypertensive patients, analyzing seven randomized clinical trials. The results showed a small, statistically significant reduction in SBP (MD −3.00; SMD −0.28; *p* < 0.0001), but no significant impact on DBP (MD −0.70; SMD −0.07; *p* > 0.2). Despite statistical significance, SBP reduction was not clinically relevant compared with pharmacological treatments or other interventions such as sodium reduction. HIIT appears to provide modest, time-effective cardiovascular benefits, but its independent efficacy for managing hypertension is limited [90].

Coretti et al. 2024 showed that HIIT is a highly time-effective exercise method for improving cardiovascular (CV) health, metabolic function, and overall physical performance. They synthesized findings from 20 clinical trials to assess the impact of HIIT on ANS. Heart rate variability (HRV), a common marker for ANS function, showed varied adaptations to HIIT. Some studies have reported increased parasympathetic activity, while others have observed changes in sympathetic tone, suggesting that individual factors and specific HIIT protocols influence outcomes. In addition to HRV, additional CV parameters, such as blood pressure, cardiac output, and vascular resistance, revealed complex interactions between the sympathetic and parasympathetic systems [91]. Results indicate that HIIT can significantly improve ANS function, with notable improvements in autonomic CV modulation reported in diverse populations, including sedentary individuals, athletes, and those with medical conditions. The study found a better balance between sympathetic and parasympathetic activities following HIIT interventions [91].

HIIT involves short bursts of intense effort alternated with recovery periods, effectively improving both aerobic and anaerobic systems. It is typically performed at 70–90% of VO_2_peak or 85–95% of maximum heart rate, offering time-efficient benefits for cardiovascular fitness and metabolic health (Figure 1). Research has shown HIIT to be as effective or superior to MICT in improving physiological markers, particularly in populations with obesity, DM2, and cardiovascular disease. HIIT stimulates significant cardiovascular adaptations by pushing the body into high-exertion zones. However, it also increases ROS, potentially contributing to OS and inflammation. Some studies report enhanced antioxidant defenses following HIIT, suggesting a hormetic effect that may balance ROS production over time. Nevertheless, excessive or poorly managed HIIT can elevate muscle damage and systemic inflammation, as seen in highly trained athletes. Combining HIIT with MICT (COMB training) may optimize fat oxidation and improve body composition while reducing overtraining risk. Meta-analyses indicate HIIT’s modest but statistically significant effects on SBP and ANS function. Overall, HIIT is a powerful, adaptable tool with potential benefits and risks, requiring careful individualization and further research on long-term safety (Table S3).

### 4.2. Dietary Intervention

MetS is closely linked to lifestyle factors, and adopting a healthy eating pattern is an important key in treating its risk and preventing CV complications. Effective interventions focus on balancing calorie intake and expenditure. Guidelines suggest a body weight baseline reduction between 7% and 10% within 12 months through calorie deficit and physical activity. The aim is to achieve and sustain a body mass index under 25 kg/m^2^ [92].

Caloric restriction refers to a general reduction of energy intake without specific nutrient deficiencies. Studies show that a caloric restriction of 50% in rats during gestation has resulted in hypertension and insulin resistance in adults, whereas a deficit of 30% to 70% increased blood pressure [93].

Eating behavior is a significant, modifiable factor influencing the risk of MetS. Dietary modifications effectively prevent and manage this syndrome, but the optimal dietary pattern for reducing its impact remains unclear. Nutrients and specific food items can significantly impact insulin resistance and the components of MetS. Beneficial effects are associated with proteins, fiber, monounsaturated fatty acids (MUFAs), and polyunsaturated fatty acids (PUFAs) [94].

Evidence suggests that a lower intake of fast food, sugary drinks, salty snacks, sweets, and red meat can reduce the risk of MetS [84]. Certain micronutrient deficiencies also play a role in MetS development. Vitamin D deficiency has been linked to elevated risks of type 1 diabetes, CVD, hypertension, hyperglycemia, and other metabolic disorders. Nutritional patterns in individuals with this syndrome often show higher carbohydrate and lower fat intake compared to healthy individuals. Diets rich in unsaturated fats, such as omega-3 fatty acids, and low in saturated fats are associated with reduced MetS risk. Insufficient carbohydrate and protein intake has also been observed, contributing to the condition’s prevalence, especially among females [95].

According to Castro-Barquero et al. 2020, an optimal dietary pattern for treating MetS has not yet been established [86]. However, studies show that dietary modifications, such as improving food quality and adjusting macronutrient distribution, have beneficial effects on MetS and its components. Among restricted and low-fat diet interventions, the Mediterranean diet (MedDiet) and Dietary Approaches to Stop Hypertension (DASH) diet emerge as leading models for MetS prevention and treatment [96]. These dietary patterns provide balanced macronutrient distribution and emphasize nutrient-dense, high-quality foods, enabling health professionals to offer accessible dietary guidance without relying on strict restrictions.

Macronutrients play a significant role in the management of MetS. Studies show that high-glycemic index carbohydrates promote insulin resistance and increase the risk of developing DM2 in individuals. Low-glycemic index diets, which are higher in fiber, enhance satiety, reduce insulin resistance, and lower DM2 risk, making them a suitable dietary approach for MetS patients. Diets rich in MUFAs improve lipid profiles and insulin sensitivity compared to those high in saturated fatty acids (SFAs). A healthy dietary pattern emphasizes limiting saturated and trans fats, added sugars, and sodium [86].

The primary treatment objectives for MetS are achieving weight loss and reducing insulin resistance. A controlled energy diet combined with moderate physical activity can effectively improve the syndrome parameters. Specific dietary interventions do not suit all individuals. Current evidence favors healthy food-based dietary interventions over high-calorie restriction or isolated nutrient-focused diets; dietary patterns such as the MedDiet have been shown to enhance insulin resistance and manage MetS [97].

In a 2024 narrative review, Rathor and Ch analyze the powerful influence of dietary interventions on brain metabolism and neurological disorders. Dietary factors such as caloric restriction, ketogenic diets, and high-fat and high-fiber diets play a significant role in modulating metabolism and may be essential in the management of Alzheimer’s disease (AD) and Parkinson’s disease (PD). The authors emphasize the complexity of these neurological disorders, which are influenced by genetic and environmental factors, and suggest that dietary approaches may open up new treatment opportunities. They also emphasize the potential of diets rich in omega-3 fatty acids and polyphenols for neuroprotection due to their anti-inflammatory and antioxidant defense against ROS [98].

Song et al. 2022, in a national cross-sectional study, investigated the prevalence of MetS and its association with dietary patterns among 40,909 Chinese middle-aged and elderly individuals. The prevalence of MetS was found to be 37.1%. Using cluster analysis, three distinct dietary patterns were identified: diversity, northern, and southern. Participants adhering to the southern pattern exhibited a significantly lower risk of MetS, central obesity, low HDL-cholesterol, and elevated blood pressure, but a higher risk of elevated fasting glucose. Greater adherence to a diverse dietary pattern was also associated with reduced risks of central obesity and elevated blood pressure. Multivariate logistic regression models were applied to adjust for potential confounders and assess the strength of these associations. The findings emphasize the need for region-specific dietary interventions to effectively prevent and manage MetS in the Chinese middle-aged and elderly population [99].

Kim et al. in a 2022 study examined the association between empirically identified dietary patterns and the risk of MetS in adults aged 40 years or older. A total of 11,305 participants, initially free of MetS, were followed for 58,318 person-years. Dietary patterns were derived through factor analysis of 37 food groups from a validated 106-item food frequency questionnaire, followed by hierarchical clustering. Three major dietary patterns were identified separately for men and women: “vegetables/seaweeds”, “meat/poultry/seafood”, and “non-traditional/non-staple foods”. The “non-traditional/non-staple foods” pattern was inversely associated with MetS risk in both sexes, while associations for the other two patterns varied by gender. Notably, a cluster of women with a high “vegetables/seaweeds” score exhibited a higher MetS incidence, possibly due to excessive sodium intake. These findings suggest that while certain dietary patterns may protect against MetS, others may increase risk depending on food composition and gender differences [100].

Tsygankova et al. 2023 conducted a prospective clinical-epidemiological study that assessed the dietary patterns of 1600 residents of Kemerovo Oblast, Russia, to identify food stereotypes associated with cardioprotective nutrition. Factor analysis using the principal components method revealed distinct dietary patterns among the participants. Adherence to a fruit-and-vegetable dietary stereotype was unexpectedly associated with increased risks of obesity (by BMI and waist circumference) and DM2. In contrast, the protein-and-carbohydrate dietary pattern was linked to significantly lower risks of obesity, diabetes, hypercholesterolemia, and hypertriglyceridemia. Over three years of follow-up, five major dietary stereotypes emerged: vegetable, protein-and-carbohydrate, fruit, dairy, and mixed patterns. These findings highlight the dynamic nature of dietary behaviors and suggest that not all traditionally “healthy” patterns, such as high fruit and vegetable intake, are inherently cardioprotective without considering factors like caloric content and physical activity [101].

#### 4.2.1. Mediterranean Diet

MedDiet, created by Ancel Keys in 1960, is one of the most extensively studied and globally recognized dietary patterns, recognized by UNESCO as an intangible cultural heritage. The MedDiet has numerous health benefits. Observational studies highlight its positive impact on cardiovascular disease (CVD) incidence, DM2, MetS, obesity, cancer, and cognitive decline [102].

The diet includes nutrient-dense foods, characterized by foods rich in fiber from complex carbohydrates, PUFAs with antiatherogenic and anti-inflammatory properties, and bioactive compounds with antioxidative effects, including flavonoids, phytosterols, terpenes, and polyphenols. Its abundant micronutrients, such as vitamins and minerals, have positive effects on malnutrition prevention and immunodeficiencies [94].

**Figure 2:**
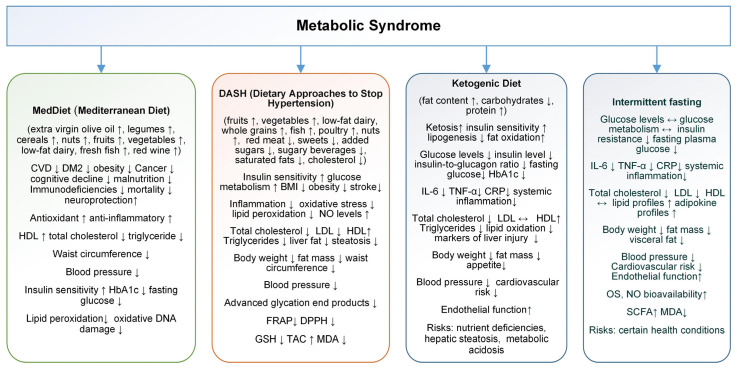
Effects, benefits, and risks of Mediterranean diet (MedDiet), Dietary Approaches to Stop Hypertension (DASH), ketogenic diet (KD), and intermittent fasting (IF) in metabolic syndrome (MetS) management (increase↑, decrease↓).

The MedDiet is one of the most studied dietary patterns worldwide. The MedDiet is recognized as a simple and healthy eating pattern with anti-inflammatory benefits. Characterized by the consumption of foods like extra virgin olive oil, legumes, cereals, nuts (e.g., walnuts), fruits, vegetables, low-fat dairy, fresh fish, and red wine, the MedDiet is increasingly linked to various health benefits (Figure 2). These foods are rich in phytonutrients, particularly polyphenols and vitamins, contributing to the MedDiet’s antioxidant and anti-inflammatory properties [103,104]. Studies show a reduced risk of coronary heart disease, stroke, vascular brain damage, and memory loss for DM2 patients; its unsaturated fatty acids improve lipid metabolism and insulin resistance, lowering overall and cardiovascular mortality rates [105,106].

The concept of the cardioprotective MedDiet originated from the Seven Countries Study, an international investigation launched in 1958 that analyzed diet and CVD in nearly 13,000 men from seven countries, including Greece, Italy, Japan, Finland, the former Yugoslavia, the Netherlands, and the United States. The study found that cardiovascular disease could be prevented by the composition of dietary fats [107].

Other subsequent studies support this conclusion. The Lyon Diet Heart Study, conducted in the 1990s, involved 605 individuals with a recent heart attack. Participants selected were divided into two groups: one followed a standard heart disease diet, while the other adopted a Mediterranean-style diet rich in omega-3-rich rapeseed oil. A 70% mortality reduction was shown for the group following the MedDiet, demonstrating the significant impact of dietary intervention on health outcomes [108].

Research consistently supports that adherence to the MedDiet can promote a healthy lifestyle and help prevent chronic diseases. For example, a systematic review found inverse associations between adherence to the MedDiet and obesity-related conditions, while a meta-analysis showed improved cardiovascular health outcomes, including reduced rates of ischemia and heart diseases [109,110].

Multiple studies confirm that the MedDiet protects against conditions such as DM2, cognitive impairment, certain cancers, aging disorders, and MetS. Furthermore, greater adherence to the MedDiet is associated with a 23% reduction in all-cause mortality, improving lipid profiles, blood pressure, and blood sugar, and reducing obesity rates (Figure 2) [109,110,111,112].

In a study, Gómez-Sánchez et al. explored the connection between the MedDiet and MetS among Caucasian adults aged 35–74. The study found that only 38.6% of the participants adhered well to the MedDiet, while 41.6% had MetS. After adjusting for confounding factors, results showed an inverse relationship between MedDiet adherence and MetS components. Specifically, greater MedDiet adherence was linked to lower systolic and diastolic blood pressure, reduced fasting glucose and triglyceride levels, and smaller waist circumference. Interestingly, higher adherence to the MedDiet was positively associated with HDL-c [113]. Logistic regression analysis demonstrated that increased MedDiet adherence reduced the likelihood of MetS and its components, except for HDL-c, which showed an opposite association. These effects were consistent across men and women, suggesting the MedDiet’s protective role in metabolic health across sexes. The study highlights MedDiet’s potential in reducing MetS risk yet calls for further research to confirm these results in non-Mediterranean populations [113].

Milano et al. 2022 in a systematic review and meta-analysis, evaluated the impact of the MedDiet compared to a low-fat diet on MetS outcomes, focusing on high-risk groups in non-Mediterranean countries. This study incorporated data from 13 RCTs, with a total of 1921 participants. MedDiet significantly reduced total cholesterol (by approximately −7.97 mg/dL) and SBP (by −2.04 mmHg) compared to a conventional low-fat diet. These reductions are particularly important because high cholesterol and elevated SBP are major risk factors for cardiovascular diseases, a common comorbidity of MetS [114]. For other components of MetS, including body weight, waist circumference, DPS, blood glucose, triglycerides, LDL, and HDL-c, the MedDiet did not show statistically significant superiority over the low-fat diet. Despite this, adherence to the MedDiet was higher among participants in non-Mediterranean regions, as assessed by the MEDAS 14-item questionnaire, indicating a preference for this dietary approach [114].

A systematic review and meta-analysis of 58 observational studies that examined the impact of adherence to the MedDiet on MetS components was conducted by Bakaloudi et al. in 2021. Findings suggest that higher adherence to the MedDiet is associated with improved parameters for MetS, particularly waist circumference, TG, and HDL-c. Specifically, individuals with high adherence to the MedDiet had significantly lower waist circumference (WC) (SMD: −0.20) and TG levels (SMD: −0.27), as well as higher HDL-c levels (SMD: 0.28). Interestingly, there was no significant difference in fasting blood glucose and SBP between high and low MedDiet adherence groups, indicating a more selective impact of the MedDiet on certain MetS components [115]. This positive association with WC, TG, and HDL may highlight the MedDiet role in reducing central obesity and improving lipid profiles, two key factors in metabolic health (Figure 2). While these findings reinforce MedDiet potential benefits, the authors emphasize the need for more research to confirm these associations and to clarify its effects on other MetS parameters such as fasting blood glucose and SBP, especially across diverse populations [115].

MedDiet is a globally recognized dietary pattern known for its extensive health benefits. It is rich in fiber, polyunsaturated fatty acids, antioxidants, and micronutrients that support cardiovascular health, prevent malnutrition, and reduce inflammation. The MedDiet has been linked to a lower risk of chronic conditions such as MetS, CVD, DM2, obesity, cognitive decline, and certain cancers. The diet’s cardioprotective effects were first highlighted in the Seven Countries Study, which found a strong association between dietary fats and cardiovascular risk. Subsequent studies, like the Lyon Diet Heart Study, demonstrated significant reductions in mortality among individuals following a MedDiet. Research consistently supports that adherence to the MedDiet improves metabolic health, including lipid profiles, BP, and blood sugar regulation. Studies have also shown that greater MedDiet adherence is associated with reduced obesity and lower rates of all-cause mortality. Furthermore, it has been shown to protect against MetS by improving components such as WC, triglycerides, and HDL cholesterol. Despite evidence of the MedDiet’s effectiveness, further research is needed to confirm its impact across diverse populations. Overall, the MedDiet remains one of the most effective and accessible dietary approaches for promoting long-term health and preventing chronic diseases.

#### 4.2.2. Dietary Approaches to Stop Hypertension

The DASH plan promotes the consumption of fruits, vegetables, low-fat dairy, whole grains, fish, poultry, and nuts while limiting red meat, sweets, added sugars, sugary beverages, saturated fats, and cholesterol. This diet is designed to increase the intake of potassium, calcium, magnesium, protein, and fiber. The DASH diet has significant effects on lowering both systolic and diastolic blood pressure in adults. Its benefits extend beyond blood pressure control, with studies suggesting improvements in MetS, insulin sensitivity, BMI, triglycerides, inflammation, and OS (Figure 2) [116,117,118,119].

Lv et al. 2024 confirm that the DASH diet significantly protects against MetS and its key components, such as hypertension, blood glucose levels, central adiposity, and unfavorable lipid profiles. This nutritional balance is essential for regulating blood pressure, improving glucose metabolism, preventing obesity, and achieving a healthier lipid profile, with significant reductions in total cholesterol and LDL-c. Additionally, the DASH diet has proven effective in normalizing blood pressure and addressing insulin resistance, which are crucial factors in managing MetS [119].

Sangouni et al. 2024, in an original study, investigated the effect of the DASH diet on fatty liver and other cardiovascular risk factors in people with MetS. A total of 60 subjects with MetS were randomized into the intervention group (DASH diet) or the control group (healthy diet). Outcomes measured included fatty liver index (FLI), hepatic steatosis index (HSI), WC, weight, BMI, and lipid profiles. After the intervention, significant improvements were observed in the DASH diet group compared to the control group, particularly in terms of FLI, HSI, WC, weight, BMI, and lipid levels. Specifically, reductions in triglycerides, total cholesterol, and LDL-c were significant in the DASH group [120]. The DASH diet also led to a significant decrease in blood pressure, both systolic and diastolic, compared to the control diet. These results suggest that the DASH diet is more effective than a standard healthy diet in managing fatty liver and cardiovascular risk factors [120].

Filippou et al. 2024 conducted an original study to compare the cardiometabolic effects of the DASH diet and the MedDiet, both with salt restriction, in subjects with grade 1 hypertension. Subjects were randomly assigned into four groups: control group (CG), salt-restricted group (SRG), DASH salt-restricted group (DDG), and MedDiet salt-restricted group (MDG). After a three-month intervention, both DDG and MDG showed significant reductions in the odds of MetS [0.29 (0.12, 0.72) and 0.15 (0.06, 0.41), respectively], with MedDiet demonstrating superior results in reducing blood pressure. In addition, all intervention groups experienced reductions in total cholesterol, LDL-c, fasting glucose, HbA1c, and systolic/diastolic blood pressure compared to the CG. The results suggested that when combined with salt restriction, MedDiet was more effective in reducing blood pressure, while DASH and MedDiet reduced MetS prevalence to similar degrees. The study emphasizes the importance of adopting effective dietary models, such as DASH and MedDiet, in the prevention and management of MetS [121].

Valenzuela-Fuenzalida et al. 2024 conducted a systematic search in several databases using the keywords “Metabolic syndrome”, “X syndrome”, “Dash dietary”, and “Dash diet”. Six studies that met the inclusion criteria were selected for this meta-analysis. All studies comparing the DASH diet with other dietary modalities reported significant improvements in various metabolic parameters. Specifically, the DASH diet was found to significantly reduce SBP [(standardized mean difference (SMD) = −8.06, CI = −9.89 to −7.32, *p* < 0.00001)], DBP (SMD = −6.38, CI = −7.62 to −5.14, *p* < 0.00001), increase HDL-c (SMD = 0.70, CI = 0.53 to 0.88, *p* < 0.00001), and decrease LDL-c (SMD = −1.29, CI = −1.73 to −0.85, *p* < 0.00001). The DASH diet is beneficial in improving altered metabolic parameters in patients with MetS. The DASH diet is a promising dietary approach for managing the symptoms and improving the general health of individuals with MetS, and its benefits should be emphasized in clinical practice [122].

Daneshzad et al. 2022 examined the impact of the DASH diet on sleep, mental health, and hormone levels in Iranian women with DM2. The RCT included 66 diabetic women, divided into two groups: one following the DASH diet and the other a control diet. Over three months, the researchers measured sleep quality and mental health using the Depression, Anxiety, and Stress Scale and hormone levels, including testosterone and follicle-stimulating hormone (FSH). Blood glucose levels (both fasting and postprandial), HbA1c, and advanced glycation end products (AGEs) were also assessed. Results showed significant decreases in HbA1c, FSH, and AGEs in the DASH group compared to the CG. In particular, testosterone and 2 h postprandial glucose (2hPPG) levels decreased significantly in the DASH group. Mental health improvements were also observed, with reductions in depression, anxiety, and stress scores. In addition, sleep quality improved, as evidenced by increased nighttime sleep duration in the DASH group. In conclusion, the DASH diet may benefit women with DM2 by lowering blood glucose, improving mental health, and improving sleep quality [123].

In an RCT study, Valipur et al. examined the effects of the DASH diet on insulin resistance, inflammation, and oxidative stress in pregnant women with gestational diabetes mellitus (GDM). Thirty-two women with GDM between 24 and 28 weeks of gestation were assigned to either a DASH or a CG for four weeks. The DASH diet, rich in fruits, vegetables, whole grains, and low-fat dairy products, aimed to reduce the intake of saturated fat, cholesterol, and sodium. After four weeks, the DASH diet group showed significant improvements compared with the GC: FPG decreased by 7.62 mg/dL, serum insulin levels decreased by 2.62 µIU/mL, and the HOMA-IR score (a marker of insulin resistance) decreased by 0.8. In addition, the DASH group showed significant increases in total antioxidant capacity (TAC) and GSH levels, indicating improvements in oxidative stress markers. No significant difference in serum CRP was observed between the groups. The study concluded that the DASH diet may be beneficial for insulin resistance and oxidative stress in pregnant women with GDM [124].

Lopes et al. investigated how the DASH combined diet (DASH-CD) affects blood pressure and OS in obese individuals with grade 1 hypertension compared with lean individuals with normal blood pressure. In the study, 12 obese hypertensive participants and 12 lean normotensive participants followed their usual diet, DASH-CD, and a low-antioxidant diet for four weeks each; the diets were assigned in a randomized order. To assess acute OS, participants underwent a 4 h infusion of intralipid/heparin. DASH-CD significantly lowered blood pressure in obese hypertensive participants by an average of 8.1/7.4 mm Hg, whereas blood pressure remained stable in lean normotensives on DASH-CD but tended to increase on the low-antioxidant diet. In addition, FRAP activity was initially higher in lean than in obese individuals on their usual diets. However, after DASH-CD, FRAP levels increased in the obese group, equaling those of the lean group [125]. During the low-antioxidant diet, markers of OS (f2-isoprostanes) increased after intralipid and heparin infusion in both groups but not when obese hypertensive participants followed DASH-CD. The findings suggest that DASH-CD may contribute to lower blood pressure and improve antioxidant capacity in obese hypertensive patients by addressing the imbalance between oxidative stress and antioxidant defense mechanisms [125].

Asemi et al. specifically examined the effects of the DASH diet on biomarkers of oxidative stress in overweight and obese women with polycystic ovary syndrome (PCOS). In this randomized controlled trial, 48 women were assigned to either a DASH or a control diet group, both calorie-restricted, for 8 weeks. Women on the DASH diet showed significant improvements in oxidative stress markers. TAC levels increased by an average of +98.6 mmol/L in the DASH group, while the CG showed a decrease of −174.8 mmol/L. Similarly, levels of GSH increased by +66.4 μmol/L in the DASH group, compared with a decrease of −155.6 μmol/L in the CG. These changes suggest that the DASH diet may counteract oxidative stress by increasing the body’s antioxidant defenses, thereby potentially reducing oxidative damage in women with PCOS. By increasing TAC and GSH, the DASH diet could help balance oxidative and antioxidant mechanisms, providing a beneficial effect on overall oxidative stress status [126].

Pirouzeh et al. 2020 have conducted a review and meta-analysis aimed at assessing how the DASH diet influences OS markers. A thorough search of multiple databases found eight studies that met the inclusion criteria, with a total of 317 participants. The analysis showed that following the DASH diet led to a significant decrease in MDA levels, a marker of oxidative damage, and a significant increase in GSH, an important antioxidant. However, there were no significant effects on NO. The DASH diet may improve TAC levels, with a potential improvement in oxidative stress parameters like F2-isoprostanes and FRAP. OS is linked to several CV risk factors, including endothelial dysfunction, and the DASH diet has been shown to positively influence endothelial function and reduce cardiovascular risk. In addition, the DASH diet’s reduction of MDA and its beneficial impact on GSH levels support its role in managing oxidative stress and improving overall health (Figure 2) [127].

Bahrami et al. 2022 demonstrated that the DASH diet is associated with improvements in oxidative stress and antioxidant capacity. Enrolled in the study, 155 young women were evaluated for adherence to the DASH diet and its effects on OS markers. Participants with the highest adherence to the diet consumed more fruits, vegetables, nuts, legumes, and low-fat dairy and had a lower intake of sugar-sweetened and sodium-sweetened beverages. The study measured oxidative stress using the FRAP and α-diphenyl-β-picrylhydrazyl (DPPH) reduction methods, as well as MDA levels. Results indicated that women with the highest adherence to the DASH diet had significantly lower levels of urinary FRAP, DPPH, and MDA compared to those with the lowest adherence. Logistic regression showed that higher adherence was related to lower markers of oxidative stress, with likelihood ratios indicating reduced urinary FRAP (0.82), DPPH (0.47), and MDA (0.13). These results suggest that the DASH diet contributes to reducing oxidative damage and improving TAS in healthy women. This study supports the DASH diet as a potential strategy for improving OS and antioxidant defense [128].

The study of Arab et al. 2022 explored the relationship between the DASH diet and oxidative stress in women diagnosed with migraine. A total of 102 women who met the inclusion criteria were randomly assigned to either the DASH diet group or the usual dietary advice group, with the intervention lasting three months. Parameters of OS, including NO, total TAC, total oxidative status (TOS), MDA, and OS index (OSI), were measured at the start and end of the study. Results showed a significant reduction in NO and TOS and a marginally significant reduction in OSI in the DASH diet group compared to the CG. Additionally, DASH diet compliance led to significant improvements in migraine-related clinical indices, such as migraine index, headache diary result, and migraine headache index score. However, no significant changes were observed in TAC and MDA. The findings suggest that the DASH diet may serve as a beneficial complementary treatment for migraine patients (Figure 2) [129].

Larsson et al. 2016 conducted a study on the DASH diet and its association with stroke risk, suggesting that adherence to the DASH diet is related to a reduced risk of ischemic stroke. The research, conducted over 11.9 years, found that people in the highest quartile of adherence to the DASH diet had a 14% lower risk of ischemic stroke compared with those in the lowest quartile. The study followed 74,404 participants in Sweden, and data were obtained from the Swedish Men’s Cohort and the Swedish Mammography Cohort. The results showed a significant inverse relationship between the DASH diet and ischemic stroke (*p* for trend = 0.002), although there was no significant association with subarachnoid hemorrhage or a strong correlation with intracerebral hemorrhage. The potential benefits of the DASH diet for stroke prevention are attributed to its composition, which emphasizes plant-based foods, low-fat dairy, whole grains, and a reduced intake of processed meats and sweets. Similar to the Mediterranean diet, the DASH diet is rich in antioxidants, low in saturated fat, and aims to reduce blood pressure, a major risk factor for stroke [130].

The study by Niknam et al. investigated the connection between adherence to the DASH diet and stroke risk in the Iranian population, involving 194 stroke patients and 194 controls. It found that individuals in the highest quartile of DASH diet adherence had a 15% lower prevalence of stroke compared to those in the lowest quartile, although this difference was marginally significant (*p* = 0.10). After adjusting for age, sex, and total energy intake, a significant inverse association was found, with individuals in the highest adherence group having a 58% lower risk of stroke (OR: 0.48; 95% CI: 0.24, 0.96). However, once BMI was considered, the association disappeared, suggesting that obesity may play a mediating role in the relationship. This highlights the importance of BMI in understanding the effects of diet on stroke risk and indicates that while the DASH diet may offer protective benefits, obesity could attenuate its effects [131].

In a recent study, Agarwal et al. investigated the relationship between dietary patterns and AD pathology in older adults, focusing on the MIND diet and MedDiet. The MIND diet is a combination of the MedDiet and the DASH diet, designed specifically to promote brain health. The analysis included 581 participants from the Rush Memory and Aging Project, with dietary intake assessed via food frequency questionnaires and AD pathology evaluated in postmortem brain tissue. The results indicated that adherence to both diets was associated with lower global AD pathology and reduced β-amyloid load. Specifically, the MIND diet showed a significant inverse association with β-amyloid (*p* = 0.050), and the Mediterranean diet demonstrated even stronger effects (*p* = 0.004). Furthermore, individuals with a higher intake of green leafy vegetables had significantly less AD pathology compared to those with a lower intake. The study highlights the potential of diet in mitigating AD progression, particularly through its impact on β-amyloid deposition. These results suggest that MIND and MedDiet may offer neuroprotective benefits. The study also underscores the importance of dietary components like leafy greens in slowing cognitive decline. Future research should further explore the mechanisms linking diet to AD pathology [132].

OS and inflammation are significant contributors to aging and chronic disease development, making them essential targets for dietary strategies aimed at prevention. Aleksandrova et al. analyzed studies published from 2015 to 2020 and investigated the associations between dietary patterns and biomarkers of OS and inflammation. The review included 29 studies (16 observational and 13 intervention studies) and followed PRISMA guidelines, with quality assessment performed using NUTRIGRADE and BIOCROSS tools. Overall, plant-based diets, such as the Mediterranean and DASH diets, were inversely associated with biomarkers of OS and inflammation. Notably, the Mediterranean diet was linked to reduced lipid peroxidation and oxidative DNA damage, while the DASH diet was associated with lower lipid peroxidation and higher NO levels. Vegetarian, USDA Healthy Eating Index-based, and paleolithic diets also demonstrated inverse associations with oxidative and inflammatory markers [133]. Conversely, Western and fast food diets were positively associated with oxidative stress and inflammation, indicating higher levels of these harmful markers. This study concludes that plant-based dietary patterns are promising for reducing OS and inflammation, potentially supporting chronic disease prevention [133].

The DASH diet, which promotes the consumption of fruits, vegetables, low-fat dairy, and whole grains while limiting red meat and added sugars, has shown significant benefits in MetS. Recent studies confirm that the DASH diet can improve key components of MetS, including hypertension, blood glucose levels, lipid profiles, and central adiposity. Lv et al. found that the diet reduces total cholesterol and LDL-c, while improving insulin resistance and blood pressure. In addition, Sangouni et al. demonstrated the DASH diet’s superiority over a standard healthy diet in improving fatty liver and cardiovascular risk factors. Filippou et al. compared the DASH diet to the MedDiet and found both reduced the prevalence of MetS, with the MedDiet showing greater effectiveness in lowering blood pressure. The DASH diet has also been associated with improvements in OS markers, as demonstrated by studies on individuals with PCOS and obesity. Additionally, the DASH diet positively impacts mental health, sleep quality, and hormone levels in individuals with DM2 and GDM. Meta-analyses indicate that adherence to the DASH diet leads to a significant reduction in OS markers, such as MDA, and an increase in antioxidant capacity. Furthermore, the DASH diet has been linked to a decreased risk of ischemic stroke, as observed in longitudinal studies. Overall, the DASH diet is an effective, evidence-based dietary approach for managing MetS, improving OS, and reducing the risk of CVD.

#### 4.2.3. Ketogenic Diet

Among the various dietary strategies proposed to address MetS, the ketogenic diet (KD) has gained considerable attention for its ability to target the core metabolic disturbances of this condition. Initially introduced by Dr. Wilder in 1921 as a practical alternative to fasting for epilepsy management [134], the KD was later refined in 1926 by Dr. Peterman, who developed a protocol emphasizing high-fat content (fat-to-carbohydrate plus protein ratio of 4:1), minimal carbohydrates (10–15 g/day), and adequate protein (1 g/kg/day) [135]. This approach induces ketosis, a metabolic state in which ketone bodies from fat oxidation replace glucose as the primary energy source, offering numerous metabolic benefits such as improved insulin sensitivity, decreased lipogenesis, and enhanced fat oxidation—all crucial in managing MetS.

Research demonstrates that KD enhances insulin sensitivity beyond weight loss. By restricting carbohydrate intake, KD reduces monosaccharide absorption, leading to lower postprandial glucose levels, reduced pancreatic insulin demands, decreased insulin levels, and a lower insulin-to-glucagon ratio [136,137,138]. For example, a study by Battezzati et al. found that metabolizing a ketogenic meal requires nearly ten times less insulin than a Mediterranean diet meal [138]. Additionally, a meta-analysis of DM2 patients reported significant reductions in fasting blood glucose (−1.29 mmol/L), HbA1c (−1.07), triglycerides (−0.72 mmol/L), and total cholesterol (−0.33 mmol/L), with HDL-c increasing by 0.14 mmol/L over interventions lasting 1–56 weeks [139].

KDs also improve lipid profiles, reducing triglycerides and increasing HDL-c levels more effectively than low-fat diets. Studies comparing KD to diabetes-recommended diets over 3, 6, and 12 months showed KD’s superior impact on HbA1c, weight, and triglycerides, though LDL-c changes were insignificant [139]. Importantly, triglyceride reductions persisted even after 12 months, despite diminishing differences in HbA1c and weight loss between diet groups.

Interestingly, in a one-year study involving nondiabetic individuals, Brinkworth et al. [140] reported comparable reductions in fasting glucose, insulin, insulin resistance, insulin sensitivity, and CRP between individuals following an isocaloric KD (ICKD) and those adhering to a low-fat diet. The ICKD included <20 g/day of carbohydrates for the first 8 weeks and <40 g/day thereafter. Despite achieving similar weight loss, the KD group experienced a greater increase in HDL-c levels and a more substantial reduction in triglycerides, whereas the low-fat diet group exhibited lower LDL-c levels. These results align with findings from numerous randomized controlled trials, which have consistently shown that low- or very low-carbohydrate diets outperform low-fat diets (≤30% of daily energy intake) in improving cardiovascular risk factors among overweight and obese individuals [141,142].

A recent comprehensive review by Suarez et al. evaluates the effects of a very low-calorie ketogenic diet (VLCKD) on lipid metabolism, focusing on its implications for CV health, obesity, DM2, and hypercholesterolemia [143]. The VLCKD has garnered attention for its potential benefits in addressing obesity, diabetes, and dyslipidemia through its unique macronutrient composition—~44% fats, ~43% proteins, and ~13% carbohydrates—and a strict caloric restriction of <800 kcal per day. By inducing ketosis, this diet suppresses appetite, promotes weight loss, and enhances lipid metabolism.

A 2004 RCT comparing low-fat and low-carbohydrate diets in 120 obese individuals with hyperlipidemia showed that the low-carb group experienced greater reductions in triglycerides (−0.84 vs. −0.31 mmol/L) and higher increases in HDL-c (0.14 vs. −0.04 mmol/L), with no significant changes in LDL-c between groups [144].

A meta-analysis by Bueno et al. 2013 evaluating VLCKD over 12 months found greater reductions in body weight, triglycerides, and diastolic blood pressure, alongside increases in both HDL and LDL cholesterol, compared to conventional diets [145].

In DM2 research, a 2021 study showed KD increased lipid oxidation but also liver lipid content, while low-carb diets reduced triglycerides and markers of liver injury [146]. Another 2021 meta-analysis of 10 RCTs found no significant differences in HDL, LDL, or triglycerides between KD and balanced diets, though studies with more female participants noted triglyceride reductions [147]. Moreover, in another meta-analysis of 21 RCTs, Luo W et al. (2022) showed that KD was more effective in lowering triglycerides (SMD = −0.32) and body weight, with trends toward HDL improvement (SMD = 0.07), but had no significant effect on total cholesterol or LDL [148]. Overall, while VLCKD offers significant short-term benefits for weight loss and cardiovascular risk factors, its long-term safety, sustainability, and adherence require further study, emphasizing the need for personalized medical supervision.

The KD has demonstrated significant benefits across the key components of MetS. First, in terms of obesity and body composition, KD has consistently been shown to promote weight loss and improve body composition, making it particularly effective in individuals with obesity, a primary risk factor for MetS. This effect is largely due to increased fat oxidation, appetite suppression, and the preservation of lean muscle mass. A meta-analysis on the subject revealed that KD outperforms traditional low-fat diets in inducing weight loss. Furthermore, Paoli et al. 2020 found that overweight women with PCOS, a condition closely associated with MetS, experienced a mean weight loss of 9.43 kg following a 12-week KD intervention [149]. These findings highlight the significant role that KD can play in addressing one of the central components of MetS—obesity.

The KD shows potential in improving blood pressure by targeting key CV factors, though its direct effects on hypertension remain underexplored. Studies suggest that KD reduces blood pressure primarily through rapid weight loss, fat mass reduction, and improved cardiovascular profiles, including reductions in systemic inflammation and endothelial dysfunction. While early meta-analyses yielded mixed results, some findings indicate modest reductions in SBP (−4.81 mmHg) and DBP (−3.10 mmHg) with low-carbohydrate diets (LCDs), along with improved lipid profiles, particularly HDL-c. The diet’s indirect benefits, stemming from enhanced weight management and control of MetS components, are pivotal in BP regulation. However, the evidence is limited by small sample sizes, variable study designs, and a lack of large-scale randomized trials directly comparing KD to other dietary approaches for hypertension management [150].

Inflammatory markers, such as IL-6 and tTNF-α, are typically elevated in individuals with MetS. The anti-inflammatory effects of KD have been well documented, with studies reporting significant reductions in these inflammatory cytokines in response to the diet [151]. By lowering these markers, KD helps to address one of the key pathophysiological factors contributing to MetS.

Despite its promising benefits, the KD is not without risks. Common concerns associated with KD include nutrient deficiencies, particularly in vitamins and minerals such as magnesium, calcium, and potassium, as well as gastrointestinal discomfort (e.g., constipation, diarrhea). More severe risks include hepatic steatosis and metabolic acidosis, which may occur with prolonged adherence to the diet, particularly in individuals with certain genetic predispositions or underlying health conditions. The restrictive nature of KD may also pose challenges to long-term adherence, which remains a significant barrier for many individuals [152,153]. These risks highlight the importance of medical supervision when implementing KD, particularly for individuals with pre-existing health conditions or those who plan to follow the diet long-term. Nonetheless, when appropriately monitored, KD remains a promising dietary intervention for managing the various components of MetS, offering significant benefits in weight loss, insulin sensitivity, lipid metabolism, blood pressure regulation, and inflammation reduction.

The KD has gained popularity for managing MetS by addressing core metabolic disturbances like insulin resistance, obesity, and dyslipidemia. KD induces ketosis, where fat oxidation replaces glucose as the primary energy source. This results in improved insulin sensitivity, reduced fat storage, and enhanced fat metabolism, critical in managing MetS. Research shows that KD reduces postprandial glucose, lowers insulin levels, and improves lipid profiles by reducing triglycerides and increasing HDL cholesterol. Studies comparing KD to low-fat diets have shown superior results in reducing fasting glucose and triglycerides, and improving insulin sensitivity. A meta-analysis on DM2 patients reported significant improvements in blood glucose, triglycerides, and cholesterol levels following KD interventions. Additionally, KD has demonstrated potential in promoting weight loss and improving body composition, particularly beneficial for individuals with obesity, a major risk factor for MetS. The diet has also been shown to reduce inflammatory markers like IL-6 and TNF-α, further improving cardiovascular health. Despite these benefits, the KD is not without risks, including nutrient deficiencies, gastrointestinal discomfort, and potential liver complications with prolonged use. Medical supervision is crucial for those on KD to manage its risks and ensure its long-term effectiveness in addressing MetS.

#### 4.2.4. Intermittent Fasting

Intermittent fasting (IF) has emerged as a potential dietary strategy for improving metabolic health. Almabruk et al. in 2024, in a review and meta-analysis, assessed the effects of IF on weight, BMI, cholesterol, BP, and glucose levels in individuals with MetS. A total of 11 studies were included from an initial pool of 6451 identified through PubMed and Google Scholar. The analysis followed PRISMA guidelines and utilized a random-effects model. IF resulted in significant reductions in weight (−3.59 kg) and BMI (−1.39 kg/m²). LDL-c levels decreased by 56.22 mg/dL, and SBP dropped by 5.54 mmHg. However, HDL-c showed minimal improvement, and glucose levels remained largely unchanged. Overall, intermittent fasting demonstrated notable benefits as a non-pharmacological intervention for managing MetS [153].

In a recent review, Vrdoljak et al. stated that IF, including time-restricted feeding (TRF), has recently gained popularity as a potential strategy to manage metabolic risk factors. IF/TRF involves limiting food intake to specific time windows, without necessarily reducing total calorie intake. Preclinical studies have shown that IF/TRF can improve glucose and insulin metabolism, lipid profiles, and gut microbiota composition and reduce body weight and visceral fat. However, the evidence from human studies is less consistent. While all human studies reported weight loss, some failed to show significant improvements in insulin resistance, BP, or cholesterol levels. The limited number of human trials and their small sample sizes reduce the generalizability of these findings [154].

IF has emerged as a promising approach, encompassing various timing methods such as alternate-day fasting and TRF. These patterns promote metabolic benefits by encouraging the switch from glucose to ketone metabolism. IF has been shown to reduce body weight and improve lipid profiles and BP. It also positively influences insulin resistance and hormone levels like leptin and adiponectin. Both preclinical and clinical studies suggest benefits of IF for conditions like obesity, DM2, and hypertension. However, the evidence from human studies is less definitive, with some trials showing IF offers no greater advantage than caloric restriction. There are concerns about applying IF in individuals with certain health conditions. Without large-scale, long-duration randomized controlled trials in people with MetS, prediabetes, and DM2, its long-term safety and efficacy remain unclear [155].

Guo et al., in an RCT, investigated the effects of IF on cardiometabolic risk factors and gut microbiota in adults aged 30–50 with MetS. A total of 39 participants were enrolled, with 21 assigned to the IF group and 18 to the control group. The IF group followed a modified “2-day” fasting protocol for 8 weeks, reducing calorie intake by 69% on fasting days. At the end of the intervention, the IF group showed significant reductions in markers of OS and endothelial function, as well as reductions in fat mass and visceral fat. Despite minimal weight loss, IF improved adipokine profiles by decreasing leptin and increasing adiponectin levels. IF also increased SCFA production and reduced levels of lipopolysaccharides, suggesting improved gut barrier integrity. Functional microbial pathways involved in carbohydrate metabolism and glycolysis were more active after IF, enhancing SCFA synthesis. Though IF did not significantly impact glucose metabolism or lipid profiles compared to controls, it reduced markers of oxidative damage, such as MDA, and improved NO bioavailability [156].

Manoogian et al. 2024, in an RCT, aimed to assess the effects of TRE on cardiometabolic health in adults with MetS. The participants, who had elevated fasting glucose or HbA1c, were randomly assigned to either standard-of-care (SOC) nutritional counseling or SOC combined with a personalized 8 to 10 h TRE regimen for 3 months. The TRE group reduced their eating window by at least 4 h compared to their baseline. The primary outcomes included HbA1c, fasting glucose, fasting insulin, and insulin resistance, measured through continuous glucose monitoring. The study found that TRE modestly improved HbA1c by −0.10% compared to SOC, with no major adverse events reported. A total of 108 participants completed the intervention, with a mean baseline age of 59 years and a body mass index of 31.22 kg/m^2^. The results were statistically adjusted for age, and the intervention did not show any significant negative side effects. Overall, TRE proved to be a practical and effective lifestyle intervention, improving glycemic control in individuals with MetS [157].

Mezhal et al. 2024, in a cross-sectional study, investigated the prevalence of MetS in young adults and assessed whether HbA1c could be a suitable glycemic marker in non-fasting individuals. Researchers used baseline data from 5161 participants, with only 21% of participants in a fasting state at the time of sample collection. The age-adjusted MetS prevalence was 22.7% in men and 12.5% in women under 40 years of age. Substituting fasting blood glucose with HbA1c did not significantly affect MetS diagnosis among fasting individuals (*p* > 0.05). Multivariate regression analysis showed strong associations between MetS and increased age, higher BMI, and a family history of metabolic or CVD. These results suggest HbA1c is a viable alternative to fasting blood glucose in assessing MetS in non-fasting populations. The study highlights a concerningly high prevalence of MetS in young adults and provides practical implications for screening practices in both fasting and non-fasting settings [158].

Parvaresh et al. 2019, in an RCT, evaluated the effects of calorie restriction (CR) versus modified alternate-day fasting (ADF) in 70 adults with MetS over 8 weeks. Participants were randomly assigned to either a personalized CR diet or an ADF regimen, both supervised by dietitians. The study assessed anthropometric, metabolic, and cardiovascular parameters before and after the intervention. Of the 70 participants, 69 completed the study. Compared to CR, the ADF group showed significantly greater reductions in body weight (*p* = 0.003), WC (*p* = 0.026), SBP (*p* = 0.029), and fasting plasma glucose (*p* = 0.009). However, there were no significant differences between the two groups in lipid profiles (triglycerides, total cholesterol, LDL-c, HDL-c), DBS, HOMA-IR, or fasting insulin levels. Both dietary interventions were well tolerated, with no reports of adherence issues. The findings suggest that ADF may offer more pronounced short-term benefits for weight and glucose control than traditional CR. However, the lack of difference in insulin resistance markers and lipid parameters indicates the need for longer studies. Overall, modified ADF appears to be a promising strategy in the management of key components of MetS [159].

IF has gained attention as a promising strategy for managing MetS, with various methods such as TRF and ADF showing benefits. Studies have demonstrated that IF can lead to weight loss, improved lipid profiles, and reductions in blood pressure, though its effects on glucose metabolism and insulin resistance are less consistent. For instance, a meta-analysis by Almabruk et al. found significant reductions in weight [153], BMI, and LDL cholesterol, while Vrdoljak et al. highlighted improvements in glucose metabolism and insulin resistance in preclinical studies but noted inconsistent results in human trials [154]. Clinical studies, such as the one by Guo et al., show improvements in oxidative stress, gut microbiota, and adipokine profiles with IF, though effects on glucose and lipid profiles were minimal [156]. Other research, like the RCT by Emily et al., found modest improvements in HbA1c with TRE, confirming its practicality as an effective lifestyle intervention [157]. Despite these benefits, concerns about long-term efficacy and safety, particularly in individuals with certain health conditions, persist. Thus, while IF appears promising, further large-scale trials are needed to confirm its long-term impact on MetS.

## 5. Concluding Remarks

MetS is a multifactorial condition resulting from the interaction of genetic, environmental, and lifestyle factors. It is characterized by central obesity, insulin resistance, hypertension, dyslipidemia, and chronic inflammation, with ANS dysregulation playing a central role in its pathophysiology. Increased SNS activity, PNS dysfunction, and chronic activation of the HPA axis exacerbate metabolic disorders such as glucose intolerance, dyslipidemia, and increased adiposity. These factors contribute to the progression of CVD and DM2, making effective management of MetS essential.

Exercise, particularly low- to moderate-intensity exercise, has been shown to be an effective strategy to manage MetS. Low-intensity exercise leads to a decrease in SNS activity, an increase in PNS activity, and a reduction in HPA axis activation, thus improving metabolism by increasing insulin sensitivity, reducing HbA1c levels, and decreasing waist circumference. In addition, low-intensity exercise decreases OS markers such as MDA and 8-OHd while stimulating the activity of antioxidants such as SOD, GPx, CAT, and TAS. Furthermore, low-intensity exercise improves lipid profiles, reduces lipid peroxides, enhances endothelial function, and lowers blood pressure, contributing to improved cardiovascular health.

Moderate-intensity exercise brings similar benefits by reducing SNS activity, increasing PNS activity, and restoring HPA axis balance. It improves insulin sensitivity, decreases HbA1c, body weight, fat mass, and WC, and increases HDL-c while reducing LDL-c and triglycerides. Inflammation is also reduced, with decreased levels of pro-inflammatory markers, such as IL-1β, IL-6, TNF-α, and IFN-γ, while anti-inflammatory markers such as IL-10 and T-helper cells increase. OS markers, including AOP, TBARS, MDA, and 8-OHdG, decrease, and endothelial function and baroreflex sensitivity improve. Moderate exercise also supports glucose tolerance, promoting better metabolic health.

HIIT offers potential benefits for MetS patients by improving metabolism and insulin sensitivity while reducing fat mass. However, it can also lead to increased oxidative stress and inflammation due to the higher production of ROS. While HIIT improves fat oxidation, endothelial function, and SBP, it may increase OS markers such as MDA and reduce levels of antioxidant enzymes like GSH and GPx. Given its intense nature, HIIT should be approached with caution and under supervision, as it may not be suitable for all MetS patients, particularly those with hypertension.

Dietary interventions, including the MedDiet and the DASH diet, have proven effective in improving MetS-related parameters. The MedDiet, which emphasizes extra-virgin olive oil, legumes, grains, nuts, fruits, vegetables, low-fat dairy, and moderate red wine consumption, is linked to a reduced risk of MetS, CVD, DM2, obesity, cancer, and cognitive decline. It improves antioxidant levels, reduces inflammation, and supports heart health by increasing HDL while reducing total cholesterol and triglycerides. The MedDiet also improves insulin sensitivity, reduces waist circumference, fasting glucose, and HbA1c, and decreases lipid peroxidation and oxidative DNA damage. On the other hand, the DASH diet focuses on increasing the intake of fruits, vegetables, whole grains, fish, poultry, and nuts while reducing red meat, sweets, added sugars, and saturated fats. It effectively reduces MetS risk, improves insulin sensitivity, glucose metabolism, and lipid profiles, and supports weight loss by reducing BMI, fat mass, and waist circumference. The DASH diet also decreases OS and inflammation, promotes reduced lipid peroxidation, and increases NO levels. Additionally, it has been shown to reduce hypertension and improve liver fat and steatosis. Both diets positively influence gut microbiota by increasing the production of SCFAs.

In addition to exercise and diet, other interventions, such as IF and KD, have been explored for their potential to manage MetS. IF can promote weight loss and improve lipid profiles but presents mixed results on glucose metabolism and insulin resistance. Further research is needed to confirm its long-term efficacy. The KD, which induces ketosis, has shown benefits in improving insulin sensitivity, reducing fat storage, and enhancing fat metabolism, leading to improved glucose control and cardiovascular health. However, it requires careful monitoring due to potential risks, including nutrient deficiencies and gastrointestinal discomfort.

Managing MetS effectively requires a personalized and comprehensive approach, combining exercise, nutrition, stress management, and gut health interventions. Prioritizing low- to moderate-intensity exercise, along with dietary strategies like the MedDiet or DASH diet, can significantly improve metabolic health. While high-intensity exercises like HIIT may offer benefits for some individuals, their safety and suitability should be assessed on a case-by-case basis. Ultimately, an integrated approach addressing lifestyle factors such as diet, physical activity, and stress management can help individuals with MetS achieve lasting improvements and reduce the risk of associated complications.

## Supplementary Materials

The following supporting information can be downloaded at: https://www.mdpi.com/article/10.3390/life15050757/s1. Table S1. Summarizing the key studies on low-intensity exercise and its effects on Metabolic Syndrome (MetS) and Oxidative Stress (OS) markers, including the types of exercise, participants, outcomes, and implications. Table S2. Summarizing the key studies on moderate-intensity exercise and its effects on Metabolic Syndrome (MetS) and Oxidative Stress (OS) markers, including the types of exercise, participants, outcomes, and implications. Table S3. Summarizing the key studies on high-intensity interval training (HIIT) and its effects on Metabolic Syndrome (MetS) and Oxidative Stress (OS) markers, including the types of exercise, participants, outcomes, and implications. Table S4. Summarizing the key studies on Mediterranean Diet (MedDiet) and its effects on Metabolic Syndrome (MetS) and Oxidative Stress (OS) markers, including the participants, outcomes, and implications. Table S5. Summarizing the key studies on Dietary Approaches to Stop Hypertension (DASH) diet and its effects on Metabolic Syndrome (MetS) and Oxidative Stress (OS) markers, participants, outcomes, and implications. Table S6. Summarizing the key studies on Ketogenic Diet (KD) and its effects on Metabolic Syndrome (MetS) and Oxidative Stress (OS) markers, including the participants, outcomes, and implications. Table S7. Summarizing the key studies on intermittent fasting (IF) and its effects on Metabolic Syndrome (MetS) and Oxidative Stress (OS) markers, including participants, outcomes, and implications.

## Abbreviations

2hPPG	2 h postprandial glucose
3-NT	3-nitrotyrosine
8-OHdG	8-hydroxy-2′ -deoxyguanosine
AD	Alzheimer’s disease
AGE	advanced glycation end products
AIM	macrophage apoptosis inhibitor
AIT	aerobic interval training
AMPK	activated protein kinase
ANS	autonomic nervous system
AOPP	advanced oxidation protein products
ARC	arcuate nucleus
ATP	adenosine triphosphate
BAT	brown adipose tissue
BMI	body mass index
CAP1	adenylyl cyclase-associated protein 1
CAT	catalase
CCK	cholecystokinin
CG	control group
CK	creatine kinase
CME	continuous moderate exercise
CNS	central nervous system
COMB	combined exercise training
CRH	corticotropin-releasing hormone
CRP	C-reactive protein
CV	cardiovascular
CVD	cardiovascular disease
DASH-CD	DASH combined diet
DASH-NP	non-plant-based DASH diet
DASH-P	plant-based DASH diet
DBP	diastolic blood pressure
DDG	dash salt-restricted Group
DM2	diabetes mellitus type 2
DMH	dorsomedial hypothalamic nucleus
DPPH	α-diphenyl-β-picrylhydrazyl
EBRE	elastic band resistance exercise
EEC	enteroendocrine cells
ENS	enteric nervous systems
FAO	Food and Agriculture Organization
FeNO	fractionated nitric oxide
FEV1	forced expiratory volume
FFQ	food frequency questionnaire
FLI	fatty liver index
FPG	fasting plasma glucose
FRAP	ferric-reducing antioxidant potential
FSH	follicle-stimulating hormone
FVC	forced vital capacity
GDM	gestational diabetes mellitus
GIP	glucose-dependent insulinotropic peptide
GLP-1	glucagon-like peptide-1
GPx	glutathione peroxidase
GRd	glutathione reductase
GSH	glutathione
HbA1c	glycosylated hemoglobin
HDL	high-density lipoprotein
HIIT	high-intensity interval training
HPA	hypothalamic–pituitary–adrenal
HRR	heart rate reserve
HRV	heart rate variability
HSI	hepatic steatosis index
ICKD	an isocaloric ketogenic diet
IDF	International Diabetes Federation
IR	insulin resistance
IF	intermittent fasting
ISAPP	International Scientific Association for Probiotics and Prebiotics
KD	ketogenic diet
LCDs	low-carbohydrate diets
LDL	low-density lipoprotein
MAPK	mitogen-activated kinase
MCP-1	monocyte chemotactic protein-1
MDA	malondialdehyde
MDG	medDiet salt-restricted Group
MetS	metabolic syndrome
MGBA	microbiota–gut–brain axis
MH-NO	metabolically healthy nonobese
MHO	metabolically healthy obese
MICT	moderate-intensity continuous training
MUFA	monounsaturated fatty acid
NO	nitric oxide
non-MHO	non-metabolically healthy obese
Nox	relationship between nitrite/nitrate
NRCTs	non-randomized controlled trials
NTS	neurotensin
OGTT	oral glucose tolerance tests
OS	oxidative stress
OSA	obstructive sleep apnea
OSI	oxidative stress index
OXM	oxyntomodulin
PBMC	peripheral blood mononuclear cells
PCOS	polycystic ovary syndrome
PD	Parkinson’s disease
PEF	peak expiratory flow
PI3K-PKB	phosphoinositide-3 kinase
PNS	parasympathetic nervous system
PPG	postprandial plasma glucose
PUFAs	polyunsaturated fatty acids
PVN	paraventricular nucleus
PYY	peptide YY
RCTs	randomized controlled trials
ROS	reactive oxygen species
SA	serum antioxidant activity
SBP	systolic blood pressure
SCFA	short-chain fatty acids
SCFAs	short-chain fatty acids
SFA	saturated fatty acids
SNS	sympathetic nervous system
SOD	superoxide dismutase
SRG	salt-restricted group
TAC	total antioxidant capacity
TAS	total antioxidant status
TBARS	thiobarbituric acid reactive substances
TRF	time-restricted feeding
TLR-4	toll-like receptor 4
TOS	total oxidative status
T-SH	total thiol content
UA	uric acid
VLCKD	very low-calorie ketogenic diet
VMH	ventromedial hypothalamus
VN	vagal nerve
VO2R	oxygen uptake reserve
WAT	white adipose tissue
WC	waist circumference
WHO	World Health Organization
