# Supplementary material for: Integrative Strategies for Preventing and Managing Metabolic Syndrome: The Impact of Exercise and Diet on Oxidative Stress Reduction—A Review
Source: Life (Basel). 2025 May 8;15(5):757. doi: 10.3390/life15050757 (PMC12113156; doi:10.3390/life15050757)
Supplement: Supplementary file 1 [file life-15-00757-s001.zip › life-3587951-supplementary.pdf]

Supplementary Materials

Table S1. Summarizing the key studies on low-intensity exercise and its effects on Metabolic Syndrome (MetS) and Oxidative Stress (OS) markers, including the types of exercise, participants, outcomes, and implications

Study	Main Focus	Key Findings	Implications	Type of Exercise	Participants	Outcomes on MetS/OS Markers
Cramer et al. (2016)	Yoga's impact on MetS	Yoga reduced waist circumference and systolic blood pressure but had no significant effect on triglycerides, HDL-c, or fasting plasma glucose.	Suggests yoga can reduce specific MetS parameters, but is not a comprehensive solution for MetS.	Yoga	794 participants in 7 RCTs	Reduced waist circumference (SMD = -0.35) and systolic BP (SMD = -0.29); no effect on triglycerides, HDL-c, or fasting plasma glucose
Khoshnaw & Ghadge (2020)	Yoga's effect on MetS risk factors	Mixed results: beneficial effects on insulin sensitivity and blood pressure, but inconsistent evidence overall.	Inconclusive evidence; more robust trials needed to confirm yoga's effectiveness for MetS risk factors.	Yoga	Systematic review of multiple studies	Improvement in insulin sensitivity and blood pressure in some studies, but mixed results overall
Patil et al. (2014)	Yoga's impact on OS markers in elderly hypertensive individuals	Yoga reduced serum MDA and increased antioxidant levels (SOD, GSH, Vitamin C), while the control group showed worsened OS markers.	Yoga is effective for reducing OS and improving antioxidant defense mechanisms in elderly hypertensive individuals.	Yoga	57 elderly males with grade I hypertension	Reduced MDA ($p<0.001$), increased SOD ($p<0.007$), GSH ($p<0.002$), and vitamin C ($p<0.002$) levels; control group worsened in OS markers.
Venugopal et al. (2022)	Yoga's effect on OS in adults with DM2	Yoga significantly reduced MDA levels and improved glycemic control (fasting plasma	Supports yoga as an adjunct therapy for reducing OS and improving glycemic	Yoga	Systematic review and meta-analysis	Reduced MDA (SMD = -1.4); reduced fasting plasma glucose (SMD = -1.87), HbA1c (SMD

		glucose, HbA1c), but no effect on SOD levels.	control in DM2 patients.			= -1.92); no effect on SOD levels
Promsrisuk et al. (2023)	Elastic band resistance + Thai yoga for blood glucose and OS	Combined exercise significantly reduced blood glucose, HbA1c, and OS markers (MDA), while increasing antioxidants (SOD, CAT) and improving lung function.	Combination of resistance exercise and Thai yoga could be beneficial for DM2 patients, addressing both metabolic and oxidative aspects.	Elastic band resistance + Thai yoga	42 DM2 patients	Reduced MDA and fasting blood glucose, increased SOD and CAT; improved lung function and reduced airway inflammation
Rosado-Pérez et al. (2021)	Tai Chi's effect on OS	Tai Chi increased SOD and CAT levels, while reducing lipoperoxides compared to sedentary behavior.	Tai Chi can improve antioxidant activity and reduce OS in older adults, offering a low-intensity exercise option for better health.	Tai Chi	Systematic review and meta-analysis of 10 studies	Increased SOD and CAT, reduced lipoperoxides compared to sedentary behavior; limited comparisons with other forms of exercise
Mendoza-Núñez et al. (2018)	Tai Chi's impact on OS and inflammatory markers in MetS adults	Tai Chi reduced OS score and HbA1c, increased TAS, but no significant changes in cardiovascular parameters like blood pressure.	Tai Chi may be an effective low-intensity intervention for improving OS and glycemic control in MetS patients.	Tai Chi	110 sedentary older adults with MetS	Reduced OS score ($p<0.05$), increased TAS, decreased HbA1c, no significant changes in blood pressure or heart rate
Chang & Liu (2021)	Resistance training, Tai Chi, and their combination on OS, glycemia	Combined Tai Chi and resistance training showed the greatest reduction in OS markers (MDA, 8-OHdG) and improved SOD, but did not improve glycemic or lipid control compared to individual interventions.	Combined exercise is more effective for reducing OS markers, but not necessarily for improving glycemic or lipid control.	Resistance training + Tai Chi	94 elderly patients with DM2	Reduced OS markers (MDA, 8-OHdG), improved SOD levels; no significant improvements in glycemic or lipid control compared to individual exercises

Rosado-Pérez et al. (2020)	Tai Chi vs walking on OS in older adults	Tai Chi reduced lipoperoxides, improved SOD, and reduced overall OS score more effectively than walking.	Tai Chi may generate a stronger antioxidant response than walking, potentially delaying aging processes.	Tai Chi, Walking	106 clinically healthy older adults aged 60-74	Reduced lipoperoxides, improved SOD, reduced OS score more effectively than walking.

Table S2. Summarizing the key studies on moderate-intensity exercise and its effects on Metabolic Syndrome (MetS) and Oxidative Stress (OS) markers, including the types of exercise, participants, outcomes, and implications

Study	Main Focus	Key Findings	Implications	Type of Exercise	Participants	Outcomes on MetS/OS Markers
Carroll & Dudfield (2006)	Impact of moderate-intensity exercise on MetS	Improved HDL-c, reduced triglycerides, increased insulin sensitivity, reduced BP.	Effective in preventing or delaying DM2 onset, addressing multiple MetS factors	Aerobic exercise (walking, cycling)	Overweight/obese adults with elevated BP	Reduction in BP, triglycerides, improved insulin sensitivity, prevention of DM2, improved HDL
Tjønna et al. (2008)	Comparison of aerobic interval vs. continuous exercise on MetS	AIT improved VO_2max and MetS risk factors more than CME, but both reduced BP	Both AIT and CME are beneficial for MetS, AIT showed superior improvements in fitness and MetS factors	Aerobic interval training, CME	32 adults with MetS	Greater VO_2max increase, reduction in MetS risk factors (fat mass, BP) with AIT, reduction in BP with both
Ostman et al. (2017)	Effects of aerobic exercise on MetS risk factors	Reduced BMI, waist circumference, BP, blood glucose, triglycerides, LDL with aerobic exercise	Aerobic exercise significantly reduces several MetS markers, beneficial for MetS management	Aerobic exercise (walking, cycling)	77,000 patient hours of exercise across multiple trials	Significant reductions in BMI, waist circumference, BP, fasting glucose, triglycerides, LDL

Lwow et al. (2011)	Exercise and oxidative stress in postmenopausal women with MetS	No significant differences in oxidative stress between MHO and non-MHO groups	Exercise may not change OS markers in MetS, but regular physical activity may help manage MetS	Aerobic exercise (walking)	161 postmenopausal women with MetS	No significant change in OS markers (AOPP, MDA) between MHO and non-MHO groups after exercise
Farinha et al. (2015)	Impact of aerobic training on inflammation and oxidative stress	Decrease in pro-inflammatory cytokines (IL-1β, IL-6) and oxidative stress markers (AOPP, TBARS)	Aerobic exercise is effective in reducing inflammation and OS in MetS	Aerobic exercise (walking, cycling)	23 women with MetS	Reduced IL-1β, IL-6, TNF-α, TBARS, AOPP, and increased antioxidant markers (IL-10, T-SH)
Poblete Aro et al. (2015)	Comparison of HIIT vs. CME on oxidative stress and inflammation	HIIT is more effective in reducing MDA, improving GPx, and NO concentrations than CME	HIIT offers superior benefits for reducing OS in DM2 patients	HIIT vs. CME	Adults with DM2 (number unspecified)	Reduced MDA, improved antioxidant levels (GPx, NO), better OS normalization with HIIT
Rytz et al. (2020)	Long-term effects of exercise on oxidative stress in older adults with MetS	Exercise reduced AOPP after 6 months; higher OS in participants with MetS	Regular exercise reduces OS markers in older adults with MetS, particularly AOPP	Aerobic exercise (walking, cycling)	206 older adults	Reduced AOPP, improved oxidative balance after 6 months of aerobic exercise
Nojima et al. (2014)	Impact of moderate-intensity aerobic training on oxidative stress and glycemic control	Reduced 8-OHdG levels, improved glycemic control, and significant reduction in oxidative stress	Aerobic training can improve oxidative stress markers and glycemic control in individuals with MetS	Aerobic exercise (walking, cycling)	43 (group A), 44 (group B), 16 (control)	Reduced 8-OHdG, improved glycemic control in aerobic training groups
Dekleva et al. (2017)	Chronic exercise effects on oxidative stress in hypertensive patients	Chronic exercise improved antioxidant defense and endothelial function	Chronic exercise helps normalize OS markers and may benefit hypertensive patients	Aerobic exercise (walking, cycling)	Hypertensive patients	Improved antioxidant defense, endothelial function, reduction in OS (AOPP, MDA)

Table S3. Summarizing the key studies on high-intensity interval training (HIIT) and its effects on Metabolic Syndrome (MetS) and Oxidative Stress (OS) markers, including the types of exercise, participants, outcomes, and implications

Study	Main Focus	Key Findings	Implications	Type of Exercise	Participants	Outcomes on MetS/OS Markers
Gibala & McGee (2008)	Comparison of HIIT vs traditional endurance training	HIIT induces similar or superior improvements in cardiovascular fitness and health markers.	HIIT is a time-efficient alternative to traditional endurance training for improving cardiovascular and metabolic function.	HIIT	Healthy individuals	Significant improvements in cardiovascular fitness and health markers (MetS) with shorter training time compared to traditional methods
de Araujo et al. (2016)	Effects of HIIT on redox balance in muscles	HIIT increases ROS production and lipid peroxidation, disrupting antioxidant enzyme function.	HIIT can lead to oxidative stress, requiring careful management of exercise intensity to avoid detrimental effects.	HIIT	Healthy adults	Elevated ROS and lipid peroxidation, disrupting antioxidant defense, indicating a complex interaction between intensity and OS
Sarkar et al. (2021)	OS and inflammation during HIIT in endurance athletes	HIIT increases muscle damage markers (CK, LDH), inflammatory markers (IL-6, TNF-α), and OS markers (MDA, SOD, GSH, GPx).	HIIT improves physical fitness but may increase muscle damage and oxidative stress, highlighting the need for supervision.	HIIT	Young male endurance athletes (n = 40)	Increased muscle damage, inflammation, and OS markers, but improved fitness (VO2max, strength). Elevated OS and inflammation suggest the potential risks of overtraining.
D'Alleva et al. (2022)	HIIT vs COMB and fat oxidation	Both HIIT and combined exercise led to reductions in body mass and fat mass, with improved VO_2peak.	HIIT is effective for fat loss and improving exercise capacity; combined exercise may further improve fat oxidation.	HIIT + COMB	Obese adult males (n = 34)	Reduction in body mass (3.09 kg) and fat mass (3.90 kg), improved VO_2peak, increased fat oxidation (0.32 to 0.36 g/min)

Li et al. (2021)	HIIT vs MICT on blood pressure in hypertensive patients	HIIT is more effective than MICT in reducing SBP during daytime monitoring and improving vascular function.	HIIT may provide superior benefits for blood pressure regulation in hypertensive individuals.	HIIT + MICT	Hypertensive patients (n = 442)	Greater reduction in SBP and improved flow-mediated vasodilation with HIIT compared to MICT. Both HIIT and MICT are equally effective in improving resting HR and VO_2max.
Romero-Vera et al. (2021)	Meta-analysis of HIIT effects on blood pressure in hypertensive patients	Small but statistically significant reduction in SBP, no significant effect on DBP.	HIIT offers modest cardiovascular benefits but may not be sufficient for managing hypertension independently.	HIIT	Hypertensive patients (7 RCTs)	Small reduction in SBP (not clinically relevant); no impact on DBP, indicating limited independent efficacy for hypertension management
Coretti et al. (2024)	Effects of HIIT on ANS function	HIIT improves cardiovascular health and ANS function, including HRV, blood pressure, and vascular resistance.	HIIT has a significant impact on cardiovascular modulation and autonomic function, enhancing sympathetic and parasympathetic balance.	HIIT	Diverse populations (sedentary individuals, athletes, and clinical conditions)	Significant improvements in HRV, cardiac output, and vascular resistance, with better balance in ANS activity

Table S4. Summarizing the key studies on Mediterranean Diet (MedDiet) and its effects on Metabolic Syndrome (MetS) and Oxidative Stress (OS) markers, including the participants, outcomes, and implications

Study	Main Focus	Key Findings	Implications	Participants	Outcomes on MetS/OS Markers
**Seven Countries Study (1958)**	Dietary fats and cardiovascular disease	Mediterranean diet associated with lower CVD incidence due	Strong evidence for the cardioprotective role of MedDiet.	13,000 men from 7 countries	Reduced CVD incidence and lower cardiovascular risk.

Study	Main Focus	Key Findings	Implications	Participants	Outcomes on MetS/OS Markers
		to beneficial composition of dietary fats (PUFAs).			
**Lyon Diet Heart Study (1990s)**	Mediterranean diet vs. standard heart disease diet	70% reduction in mortality in the MedDiet group; the diet's omega-3-rich content improved heart health significantly.	Supports MedDiet as a post-heart attack intervention to reduce mortality.	605 individuals with recent heart attack	Significant reduction in cardiovascular mortality.
**Gómez-Sánchez et al. (2019)**	MedDiet adherence and MetS components	Higher MedDiet adherence linked to lower blood pressure, reduced fasting glucose and triglycerides, smaller waist circumference.	Highlights MedDiet's role in reducing MetS components and suggests its potential as a preventive measure for MetS.	Caucasian adults aged 35-74	Lower systolic and diastolic BP, reduced fasting glucose, triglycerides, smaller waist circumference, improved HDL-c levels.
**Milano et al. (2022)**	MedDiet vs. low-fat diet on MetS outcomes	MedDiet significantly reduced total cholesterol and systolic blood pressure compared to low-fat diet; higher adherence in non-Mediterranean regions.	Emphasizes MedDiet as an effective approach for improving cardiovascular risk factors, particularly cholesterol and blood pressure.	1,921 participants (RCTs, high-risk groups)	Reduction in total cholesterol, systolic BP, but no significant difference in other MetS parameters.
**Bakaloudi et al. (2021)**	MedDiet adherence and MetS parameters	High adherence to MedDiet led to lower waist circumference, triglycerides, and higher HDL-c. No significant effects on fasting glucose and BP.	Suggests MedDiet's selective impact on certain MetS markers, particularly central obesity and lipid profiles.	58 observational studies (varied populations)	Reduced waist circumference, triglycerides, increased HDL-c, no significant changes in fasting blood glucose and BP.

Table S5. Summarizing the key studies on Dietary Approaches to Stop Hypertension (DASH) diet and its effects on Metabolic Syndrome (MetS) and Oxidative Stress (OS) markers, participants, outcomes, and implications

Study	Main Focus	Key Findings	Implications	Participants	Outcomes on MetS/OS Markers

**Lv et al. (2024)**	Effect of DASH diet on MetS components	DASH diet reduces hypertension, blood glucose, central adiposity, and unfavorable lipid profiles; improves cholesterol and insulin resistance	DASH diet helps regulate blood pressure, improve glucose metabolism, and prevent obesity	Adults with MetS	Lower blood pressure, improved glucose metabolism, reduced LDL-c, better insulin sensitivity
**Sangouni et al. (2024)**	DASH diet vs. healthy diet on fatty liver and cardiovascular risk factors	DASH diet reduces fatty liver index (FLI), hepatic steatosis index (HSI), weight, BMI, and lipid levels	DASH diet is more effective than a standard healthy diet in managing cardiovascular risks	60 subjects with MetS	Decreased FLI, HSI, weight, BMI, triglycerides, LDL-c, and BP
**Filippou et al. (2024)**	Comparison of DASH vs. MedDiet on MetS and BP	DASH and MedDiet both reduce MetS prevalence; MedDiet is more effective in BP reduction	Both diets are effective in MetS management, but MedDiet may be superior for BP control	Subjects with grade 1 hypertension	Reduced BP, cholesterol, fasting glucose, and HbA1c
**Valenzuela-Fuenzalida et al. (2024)**	Meta-analysis of DASH diet on metabolic parameters	DASH diet significantly reduces SBP, DBP, increases HDL-c, and decreases LDL-c	DASH diet is beneficial for managing MetS and improving cardiovascular health	Meta-analysis of 6 studies	Reduced SBP/DBP, increased HDL-c, decreased LDL-c
**Daneshzad et al. (2022)**	DASH diet effects on sleep, mental health, and hormone levels in women with DM2	DASH diet improves sleep quality, mental health (depression, anxiety), and hormone levels (testosterone, FSH)	DASH diet benefits include improved blood glucose, mental health, and sleep quality in diabetic women	66 Iranian women with DM2	Lower HbA1c, FSH, testosterone, and improved sleep and mental health
**Valipur et al. (2022)**	DASH diet effects on insulin resistance and oxidative stress in pregnant women with GDM	DASH diet reduces insulin resistance and improves oxidative stress markers	DASH diet is beneficial for GDM management, improving insulin sensitivity and oxidative stress	32 pregnant women with GDM	Decreased FPG, insulin, HOMA-IR, increased TAC and GSH
**Lopes et al.**	DASH diet effects on BP and OS in obese	DASH-CD lowers BP and improves antioxidant	DASH diet improves BP and oxidative stress balance in obese	12 obese hypertensive and 12 lean	Reduced BP, increased FRAP,

	individuals with hypertension	capacity in obese hypertensive patients	hypertensive individuals	normotensive participants	decreased OS markers
**Asemi et al. (2024)**	DASH diet on oxidative stress in women with PCOS	DASH diet increases TAC and GSH, reducing oxidative stress markers	DASH diet may help reduce oxidative damage and improve antioxidant defense in women with PCOS	48 overweight/obese women with PCOS	Increased TAC and GSH, reduced oxidative stress
**Pirouzeh et al. (2020)**	Meta-analysis of DASH diet on oxidative stress markers	DASH diet decreases MDA and increases GSH, improving oxidative stress parameters	DASH diet helps reduce oxidative damage and improve antioxidant defense	317 participants	Reduced MDA, increased GSH, improved oxidative stress parameters
**Bahrami et al. (2022)**	DASH diet adherence and oxidative stress	High adherence to DASH diet reduces OS markers (MDA, FRAP, DPPH)	DASH diet reduces oxidative damage and improves antioxidant defense in women	155 young women	Reduced MDA, DPPH, FRAP levels in high adherence group
**Arab et al. (2022)**	DASH diet and oxidative stress in women with migraines	DASH diet reduces NO, TOS, and OSI in migraine patients	DASH diet may complement migraine treatment by improving OS and reducing migraine severity	102 women with migraines	Reduced OS markers (NO, TOS, OSI), improved migraine indices
**Larsson et al. (2016)**	DASH diet and ischemic stroke risk	High adherence to DASH diet lowers ischemic stroke risk by 14%	DASH diet may help prevent stroke by reducing BP and promoting cardiovascular health	74,404 participants in Sweden	Reduced ischemic stroke risk, no effect on hemorrhagic stroke
**Niknam et al.**	DASH diet adherence and stroke risk in Iran	Higher adherence to DASH diet reduces stroke risk by 58%	DASH diet reduces stroke risk, with BMI as a mediating factor	194 stroke patients, 194 controls	Reduced stroke risk in high adherence group, influence of BMI

Table S6. Summarizing the key studies on Ketogenic Diet (KD) and its effects on Metabolic Syndrome (MetS) and Oxidative Stress (OS) markers, including the participants, outcomes, and implications

Study	Main Focus	Key Findings	Implications	Participants	Outcomes on MetS/OS Markers
**Battezzati et al. (2020)**	Comparison of ketogenic and Mediterranean diets	Metabolizing a ketogenic meal requires significantly less insulin than a Mediterranean diet meal	KD may improve insulin sensitivity and reduce postprandial glucose levels	Healthy adults	Reduced postprandial glucose, lower insulin demand
**Brinkworth et al. (2009)**	Comparison of isocaloric ketogenic and low-fat diets	Similar weight loss in both groups; KD led to greater HDL-c increase and triglyceride reduction	KD may provide superior cardiovascular benefits in managing MetS components like lipid profiles	Nondiabetic individuals	Increased HDL-c, reduced triglycerides, similar weight loss
**Bueno et al. (2013)**	Effects of VLCKD on lipid metabolism and weight loss	VLCKD led to greater reductions in body weight, triglycerides, and blood pressure; higher HDL and LDL cholesterol	VLCKD may offer significant short-term benefits for obesity, lipid metabolism, and cardiovascular risk factors	120 obese individuals with hyperlipidemia	Greater weight loss, triglyceride reduction, and increased HDL/LDL
**Battezzati et al. (2018)**	Effects of KD on insulin sensitivity and lipid profile	KD significantly improved insulin sensitivity, reduced triglycerides, and increased HDL-c levels	KD may be an effective approach for managing insulin resistance and dyslipidemia in MetS	Diabetic individuals (DM2)	Reduced HbA1c, triglycerides, increased HDL-c
**Suarez et al. (2021)**	Impact of VLCKD on cardiovascular health, obesity, and diabetes	VLCKD suppressed appetite, promoted weight loss, and improved lipid metabolism, including	VLCKD could be a promising intervention for managing obesity, diabetes, and hypercholesterolemia	Obese individuals with DM2 or hypercholesterolemia	Reduced triglycerides, improved lipid metabolism, weight loss

		reductions in triglycerides			
**Paoli et al. (2020)**	Effects of KD in women with PCOS (MetS-related condition)	12-week KD intervention resulted in an average weight loss of 9.43 kg in overweight women with PCOS	KD may play a significant role in addressing obesity in individuals with MetS-related conditions like PCOS	Overweight women with PCOS	Weight loss (9.43 kg), improved metabolic markers
**Luo et al. (2022)**	Comparison of KD and balanced diets in MetS management	KD more effective in reducing triglycerides and body weight, with trends toward HDL improvement	KD could be superior to balanced diets in managing triglycerides and body weight in MetS	Overweight and obese individuals	Significant reductions in triglycerides and body weight, trends in HDL
**Battezzati et al. (2019)**	Ketogenic diet impact on lipid profiles in DM2	KD reduced triglycerides, increased HDL-c, and improved insulin sensitivity compared to a low-fat diet	KD may offer more effective lipid profile improvements for diabetic patients compared to low-fat diets	Type 2 diabetic individuals	Improved insulin sensitivity, reduced triglycerides, increased HDL-c

Table S7. Summarizing the key studies on intermittent fasting (IF) and its effects on Metabolic Syndrome (MetS) and Oxidative Stress (OS) markers, including participants, outcomes, and implications

Study	Main Focus	Key Findings	Implications	Participants	Outcomes on MetS/OS Markers
**Almabruk et al. (2024)**	Effects of IF on weight, BMI, cholesterol, BP, and glucose in MetS	Significant reductions in weight (−3.59 kg), BMI (−1.39 kg/m²), LDL-c (−56.22 mg/dL), and systolic BP (−5.54 mmHg); minimal improvement in HDL-c; glucose unchanged	IF is a promising non-pharmacological intervention for MetS management, though glucose metabolism needs further research	11 studies with pooled data from 6,451 participants	Weight, BMI, LDL-c, SBP reduced; HDL-c and glucose remained unchanged
**Vrdoljak et al.**	Impact of IF/TRF on metabolic risk factors	IF/TRF improves glucose metabolism, insulin sensitivity, lipid profiles, and gut microbiota in preclinical	Need for more human studies to assess IF's effect on metabolic risk factors	Human studies with small sample sizes	Weight loss reported; inconsistent effects on insulin resistance, BP, and cholesterol

		studies; inconsistent results in humans			
**Vasim et al. (2022)**	Various IF patterns (ADF, TRF) on metabolic health	IF reduces body weight, improves lipid profiles, BP, insulin resistance, and hormone levels (leptin, adiponectin); mixed results on long-term efficacy	IF offers potential benefits for obesity, DM2, and hypertension, but long-term safety and efficacy remain uncertain	Preclinical and clinical studies	Reduction in body weight, BP, and insulin resistance; mixed effects on glucose metabolism
**Guo et al. (2024)**	Effects of IF on cardiometabolic risk factors and gut microbiota	IF reduced oxidative stress, fat mass, and visceral fat; improved adipokine profiles (decreased leptin, increased adiponectin); increased SCFA production	IF improves markers of oxidative stress and gut health; however, minimal effects on glucose metabolism and lipid profiles	39 adults with MetS, IF group (21) and control (18)	Reductions in oxidative stress markers, fat mass, visceral fat; improved adipokine profile
**Emily et al. (2024)**	Effects of TRE on cardiometabolic health in MetS	TRE improved HbA1c by −0.10% with no major adverse events; intervention was well-tolerated	TRE can be a practical and effective lifestyle intervention for glycemic control in MetS	108 adults with MetS, TRE group and SOC group	Modest improvement in HbA1c, no significant side effects
**Mezhal et al. (2024)**	Prevalence of MetS in young adults and HbA1c as a marker	22.7% of men and 12.5% of women under 40 had MetS; HbA1c can be an alternative to fasting glucose in MetS diagnosis	Highlights the need for accessible screening methods, especially in non-fasting individuals	5,161 participants (21% fasting)	High prevalence of MetS in young adults; HbA1c as a viable diagnostic marker
**Parvaresh et al. (2019)**	Comparison of CR vs. ADF in MetS management	ADF group showed greater reductions in weight ($P = 0.003$), waist circumference ($P = 0.026$), SBP ($P = 0.029$), and fasting glucose ($P = 0.009$) compared to CR	ADF may be more effective than CR for short-term improvements in weight and glucose control in MetS	70 adults with MetS, ADF group (35) and CR group (35)	Greater reductions in weight, waist circumference, SBP, and fasting glucose
